# Exploring the Role of GGA2 in Cancer Progression: Pan-Cancer Bioinformatics and Experimental Validation in Prostate Cancer

**DOI:** 10.3390/ijms27062905

**Published:** 2026-03-23

**Authors:** Yangyang Han, Ziyu Huang, Yuxuan Zou, Yunbo Zhang, Huizhen Xin, Meng Sun, Yimin Liu, Mengqi Zhang, Mengjia Li

**Affiliations:** 1 Department of Biology, School of Basic Medical Sciences, Xinjiang Medical University, Urumqi 830017, China; yyhan@xjmu.edu.cn (Y.H.); m18119180271@163.com (Z.H.); zyx0827999@163.com (Y.Z.); 18768900024@163.com (Y.Z.); xhz_2466@163.com (H.X.); sm199901@163.com (M.S.); 18199840012@163.com (Y.L.); 13894343612@163.com (M.Z.); 2 Xinjiang Key Laboratory of Molecular Biology for Endemic Diseases, Xinjiang Medical University, Urumqi 830017, China

**Keywords:** GGA2, pan-cancer, prognostic biomarker, immune, prostate cancer

## Abstract

Cancer remains a significant challenge to global public health. Preliminary studies indicate that the protein Golgi-associated, Gamma-adaptin Ear Containing, ARF Binding Protein 2 (GGA2) may influence various cancers. However, the potential role of GGA2 in oncogenesis remains unknown. We utilized data from The Cancer Genome Atlas (TCGA) and the Genotype-Tissue Expression (GTEx) projects to analyze GGA2 expression levels. Genetic variations and protein expression of GGA2 in human tissues were assessed using the cBioPortal. Gene Set Enrichment Analysis (GSEA) provided deeper insights into GGA2’s oncogenic functions. Comprehensive analysis of TCGA datasets combined with ESTIMATE and TIMER tools demonstrated significant correlations between GGA2 expression levels and clinical outcomes, survival metrics, genomic instability markers (microsatellite instability (MSI)/tumor mutational burden (TMB)), and immune microenvironment composition. Functional validation in prostate cancer models employed qRT-PCR quantification, immunoblotting verification, and cellular behavior assessments through colony formation, Transwell migration, and wound closure assays. Our findings suggest GGA2 could serve as a prognostic biomarker in various cancers. Abnormal levels of GGA2 promoter methylation and genetic alterations may contribute to its dysregulated expression in some cancers. Distinctly, GGA2 expression correlates with MSI and TMB across different cancers and is linked to the expression of immune checkpoint genes. Functionally, GGA2 is instrumental in inhibiting oncogenic mechanisms by diminishing the proliferation, colony formation, invasion, and migratory capabilities of prostate cancer cells. Our study shows that the oncogenic role of GGA2 in various cancers and GGA2 could be served as a biomarker of PARD.

## 1. Introduction

The progression of tumors is characterized by several critical aspects, such as genetic mutations, altered metabolic pathways, resistance to treatments, and reduced cellular cohesion. Cancer therapies have advanced from traditional radiation and chemotherapy to more targeted and immunotherapeutic approaches. Yet the focus on individual cancers often restricts our understanding of the broader genetic mechanisms involved, and the efficacy of immunotherapies across diverse cancers remains to be further substantiated [1]. Comprehensive databases, like the Cancer Genome Atlas (TCGA), facilitate pan-cancer studies, enabling the examination and comparison of gene behaviors across multiple cancer types. Such analyses are crucial for enhancing our understanding of diagnosis, prognosis, and immunotherapy applications [2].

In recent times, tumor immunotherapy has emerged as a promising field of research. Rather than directly destroying cancer cells, immunotherapy activates the body’s immune system to enhance its ability to recognize and eliminate tumor cells, thereby mobilizing the body’s anti-tumor immune response. Compared with traditional chemotherapy, immunotherapy has demonstrated durable and significant clinical efficacy in certain specific cancer types and patient populations, with some patients achieving long-term survival. Due to its clear therapeutic advantages in specific indications and distinct toxicity profile (mainly manifested as immune-related inflammatory reactions), immunotherapy is gradually becoming one of the important means of tumor treatment [3,4,5,6]. The tumor microenvironment (TME) plays a pivotal role in the success of cancer immunotherapy. Research has shown that the cellular makeup of the TME influences tumor growth, angiogenesis, invasion, metastasis, and resistance to chemotherapy [7]. A systematic analysis of the cellular phenotypes within the TME is essential for understanding tumor biology and optimizing immunotherapy effectiveness [8].

GGA2 (Golgi-associated, Gamma-adaptin Ear Containing, ARF Binding Protein 2), a part of the clathrin-coated vesicle adaptor protein family, engages with the epidermal growth factor receptor (EGFR) through a carboxy-terminal amphipathic alpha-helix [9]. Increasing evidence suggests that dysregulation of GGA2 plays a crucial role in various diseases, including cancer. Several studies indicate that GGA2 could be targeted for lung cancer therapy as its diminished expression is linked to disease progression [10,11]. However, the broader implications of GGA2 in pan-cancer contexts remain to be fully established.

**Figure 1:**
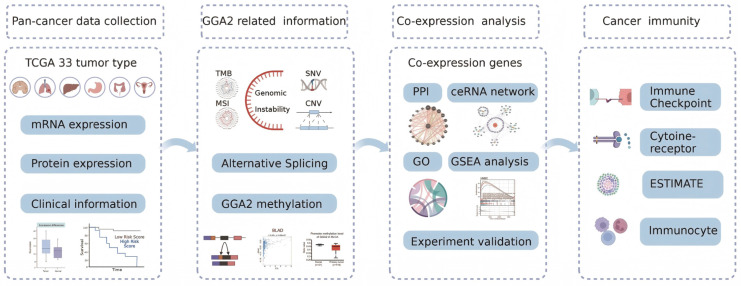
A flowchart of the study design. The flowchart describes the workflow of the study analyses.

In our research, we employed a range of integrative analytical tools and publicly available datasets of cancerous and non-cancerous tissues to perform an extensive multi-omics pan-cancer analysis of GGA2. Our objective was to elucidate its associations with clinical attributes, multi-omics variability, and specifically, its involvement in DNA repair mechanisms and cancer immunology (Figure 1). The specific biological function of GGA2 in prostate cancer has not been deeply studied. Our research team has been committed to the basic and translational research of prostate cancer. Therefore, we further explored the expression characteristics, clinical significance and potential biological functions of GGA2 in prostate cancer. We explored the biological function of GGA2 in PRAD (Prostate Adenocarcinoma) and found that GGA2 overexpression repressed cancer cell proliferation and invasion. Identifying GGA2 as a potential diagnostic, prognostic, and immunotherapeutic biomarker for various cancers marks a pivotal advancement in the field. This discovery could substantially influence the use of GGA2 in cancer immunotherapy strategies.

## 2. Results

### 2.1. Analysis of GGA2 Expression in Diverse Cancers and Normal Tissues

**Figure 2:**
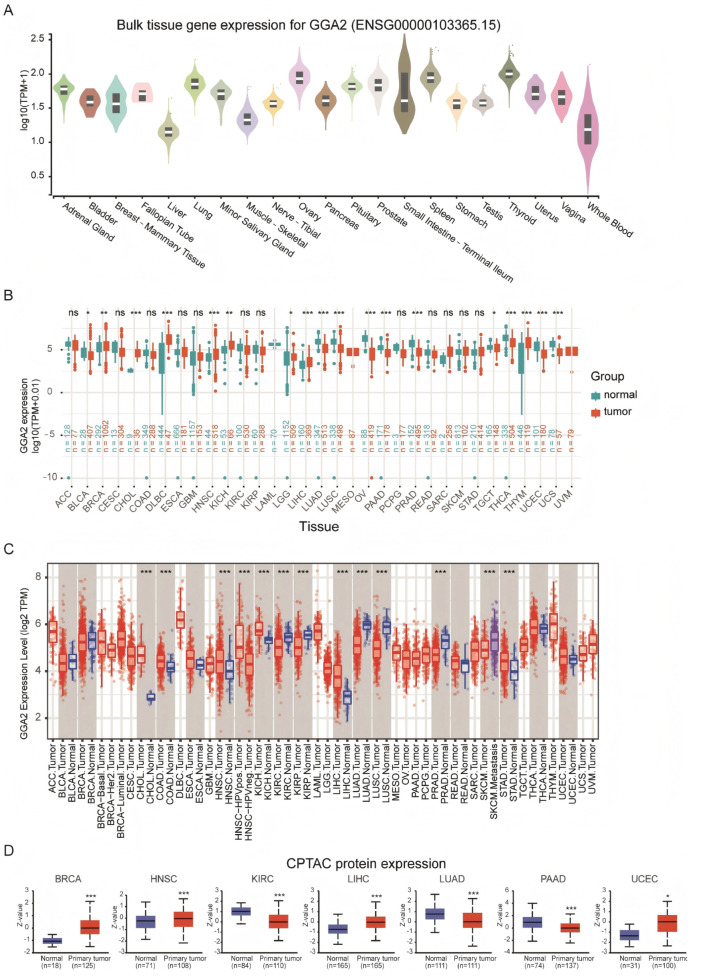
Differential expression of GGA2. (**A**) The transcriptional expression of GGA2 mRNA in normal tissues, as depicted by GTEx data. (**B**) A comparative analysis of the differential transcriptional expression of GGA2 mRNA between TCGA cancer tissues and GTEx normal tissues. The data represented by the red column pertain to cancer samples, while the blue column encompasses information related to normal samples. The normal group consists of normal tissue sourced from both the TCGA and GTEx databases. (**C**) The transcriptional expression of GGA2 mRNA across different cancer types within TIMER. (**D**) A comparative analysis of protein expression disparities between normal and primary tumor tissues of seven cancers was performed at UALCAN. * *p* < 0.05, ** *p* < 0.01, and *** *p* < 0.001. The normal group is defined as normal tissue within the TCGA database.

**Figure 3:**
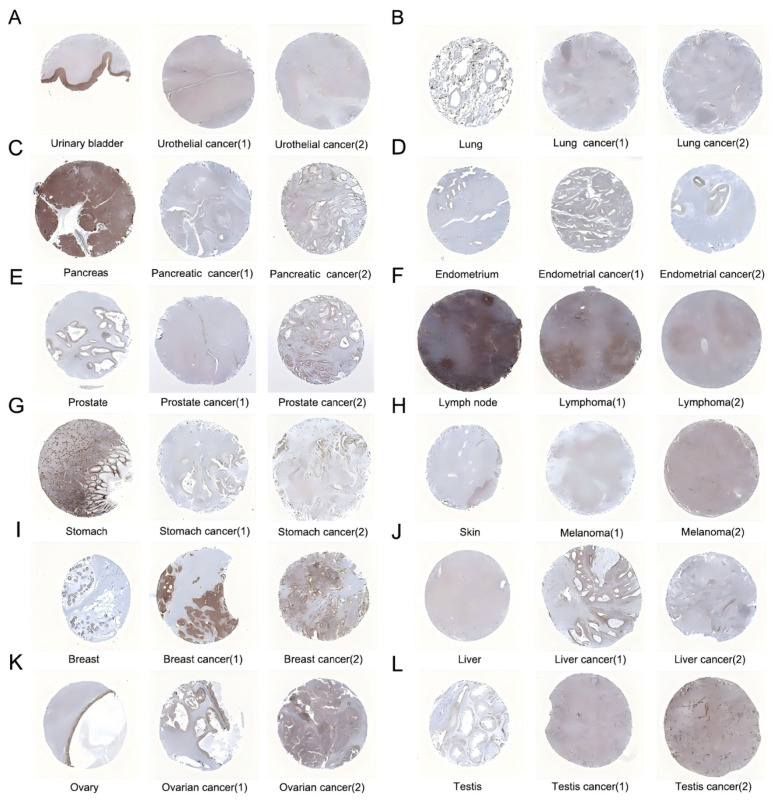
The protein expression of GGA2 in immunohistochemical images of normal (**left**) and tumor (**right**) groups. Each subgraph represents the expression of the GGA2 protein in the immunohistochemical images of different tissue types: Urothelial Carcinoma (**A**), Lung cancer (**B**), Pancreatic cancer (**C**), Esophageal cancer (**D**), Prostate cancer (**E**), Lymphoma (**F**), Stomach cancer (**G**), Skin Cutaneous Melanoma (**H**), Breast cancer (**I**), Liver cancer (**J**), Ovarian cancer (**K**), Testis cancer (**L**).

Figure 1 presents a flowchart of the study design. We explored the mRNA levels of GGA2 in standard human tissues utilizing data from the GTEx project. Prominent upregulation of GGA2 was observed in the liver, adrenal gland, and kidney tissues (Figure 2A). The study further assessed the GGA2 levels across genders in different tissues, revealing no marked differences between males and females (Supplementary Figure S1A). In addition, we surveyed the expression of GGA2 in a range of cancer types through the combined analysis of RNA Sequencing (RNA-seq) data from both TCGA and GTEx databases. We identified notable variations in GGA2 levels in 33 types of cancer, except in those where normal tissue data was absent. There was a significant increase in GGA2 levels in cancers such as breast invasive carcinoma (BRCA), cholangiocarcinoma (CHOL), diffuse large B-cell lymphoma (DLBC), head and neck squamous cell carcinoma (HNSC), kidney chromophobe (KICH), liver hepatocellular carcinoma (LIHC), testicular germ cell tumors (TGCT), and thymoma (THYM) when compared to normal controls (Figure 2B). In contrast, a reduction in GGA2 expression was found in bladder urothelial carcinoma (BLCA, *p* < 0.05), lower grade glioma (LGG, *p* < 0.05), lung adenocarcinoma (LUAD, *p* < 0.001), lung squamous cell carcinoma (LUSC, *p* < 0.001), ovarian serous cystadenocarcinoma (OV, *p* < 0.001), pancreatic adenocarcinoma (PAAD, *p* < 0.001), prostate adenocarcinoma (PRAD, *p* < 0.001), thyroid carcinoma (THCA, *p* < 0.001), uterine corpus endometrial carcinoma (UCEC, *p* < 0.001), and uterine carcinosarcoma (UCS, *p* < 0.001) relative to control tissues (Figure 2B). Furthermore, an evaluation using the TIMER database indicated a significant upregulation of GGA2 in CHOL (*p* < 0.001), colorectal adenocarcinoma (COAD, *p* < 0.001), HNSC (*p* < 0.001), KICH (*p* < 0.001), LIHC (*p* < 0.001), and stomach adenocarcinoma (STAD, *p* < 0.001), and a downregulation in renal clear cell carcinoma (KIRC, *p* < 0.001), renal papillary cell carcinoma (KIRP, *p* < 0.001), LUAD (*p* < 0.001), LUSC (*p* < 0.001), and PRAD (*p* < 0.001) compared to controls (Figure 2C). Levels of GGA2 across various cancer cell lines were also cataloged in the CCLE (Cancer Cell Line Encyclopedia) database (Supplementary Figure S1B). GGA2 protein levels were analyzed through immunohistochemistry based on the HPA (Human Protein Atlas) database, with results showing significantly lower expression in 10 cancer types and higher in 4 compared to normal tissues (Figure 3).

### 2.2. Prognostic Implications of GGA2 Expression Across Various Cancers

**Figure 4:**
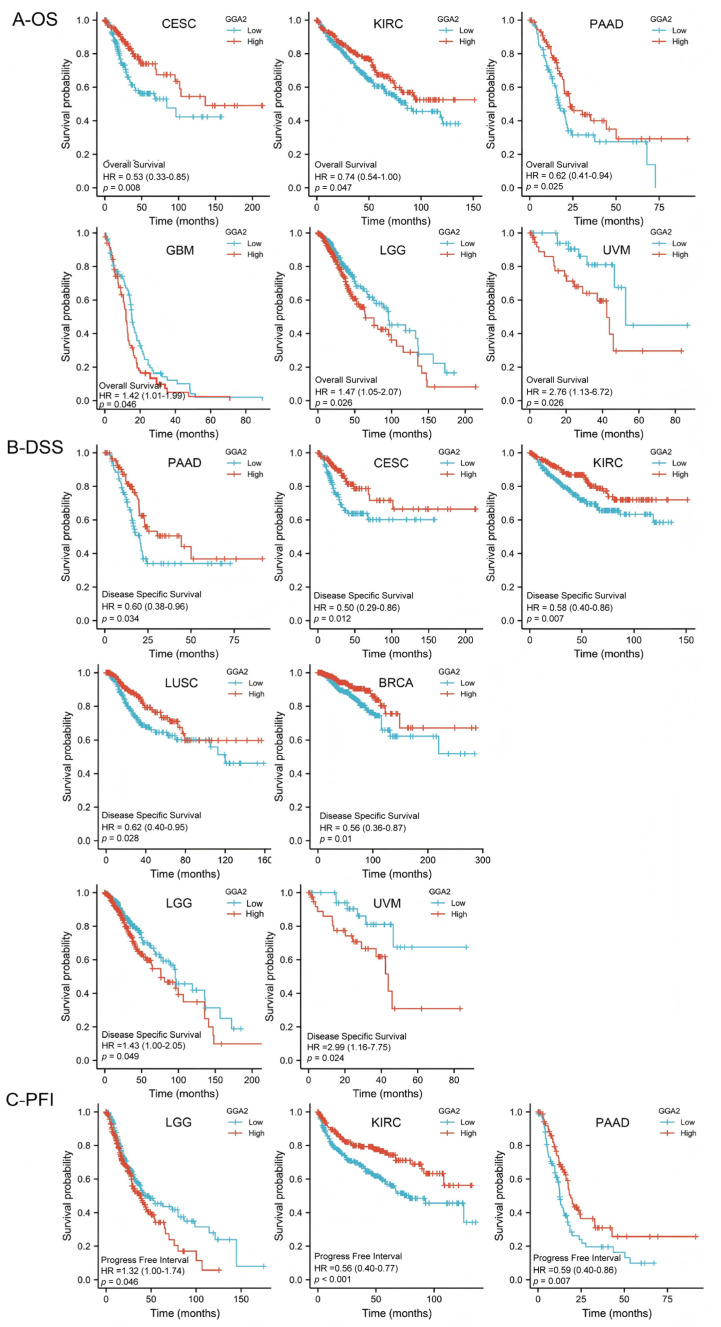
Kaplan–Meier curves are constructed with the aim of predicting the overall survival (OS), disease-specific survival (DSS), and progression-free interval (PFI) of TCGA patients. (**A**) A Kaplan–Meier analysis is performed to investigate the association between the expression of GGA2 and overall survival (OS). (**B**) A Kaplan–Meier analysis is carried out to examine the association between the expression of GGA2 and disease-specific survival (DSS). (**C**) The relationship between the GGA2 gene and progression-free interval (PFI) in TCGA patients is explored.

We further evaluated the prognostic significance of GGA2 expression. In glioblastoma multiforme (GBM), LGG, and UVM, GGA2 expression showed a significant positive correlation with overall survival (OS) (GBM: *p* = 0.046; LGG: *p* = 0.026; UVM: *p* = 0.026). Conversely, in cervical squamous cell carcinoma and endocervical adenocarcinoma (CESC), KIRC, and PAAD, GGA2 expression was negatively correlated with OS (CESC: *p* = 0.008; KIRC: *p* = 0.047; PAAD: *p* = 0.025), as shown in Figure 4A and Figure S2. Regarding disease-specific survival (DSS), GGA2 expression was identified as a protective factor in PAAD (*p* = 0.034), CESC (*p* = 0.012), KIRC (*p* = 0.007), LUSC (*p* = 0.028), and BRCA (*p* = 0.028), while it was identified as a risk factor in LGG (*p* = 0.049) and UVM (*p* = 0.024) (Figure 4B and Figure S3). Furthermore, in progression-free interval (PFI) analysis, GGA2 expression may be considered as a risk factor in LGG (see Supplementary Figure S2C), whereas it might serve as a protective factor in KIRC (*p* < 0.001) and PAAD (*p* = 0.007) (Figure 4C and Figure S4). In summary, across OS, DSS, and PFI, GGA2 expression may be identified as a risk factor in LGG, while it might emerge as a protective factor in KIRC and PAAD.

### 2.3. Examination of GGA2 Expression and Its Correlation with Clinicopathological Characteristics in Pan-Cancer

A comprehensive evaluation was conducted to explore potential links between GGA2 mRNA levels and clinicopathological characteristics across multiple malignancies. Tumor stage demonstrated statistically meaningful relationships with GGA2 abundance in five cancer subtypes ((KIRC (T1 vs. T3 *p* = 0.032), LUAD (T1 vs. T2 *p* = 0.004, T1 vs. T3 *p* = 0.002), LUSC (T1 vs. T2 *p* = 0.021), SKCM (T1 vs. T4 *p* < 0.001,T2 vs. T4 *p* = 0.074, T3 vs. T4 *p* = 0.024), STAD (T2 vs. T4 *p* = 0.047)), as detailed in Supplementary Figure S3A. Lymph node metastasis patterns in COAD (N0 vs. N2 *p* < 0.001, N1 vs. N2 *p* = 0.005), LIHC (N0 vs. N1 *p* = 0.005), and THCA (N0 vs. N1 *p* = 0.049), along with advanced pathological stage in KIRC (Stage I vs. Stage III *p* = 0.272, Stage I vs. Stage IV *p* = 0.011, Stage III vs. Stage IV *p* = 0.171) and LUAD (Stage I vs. Stage II *p* = 0.054, Stage I vs. Stage III *p* = 0.011), showed distinct associations with GGA2 expression levels (Supplementary Figure S3B). Prognostic analysis through Kaplan–Meier methodology identified diminished overall survival rates in nine cancer types (CESC (*p* = 1.6 × 10^−5^), HNSC (*p* = 0.0028), KIRC (*p* = 0.0071), LUAD (*p* = 5 × 10^−6^), LUSC (*p* = 0.017), PAAD (*p* = 0.0012), TGCT (*p* = 0.003), THCA (*p* = 0.04), thymoma) among patients exhibiting reduced GGA2 expression (*p* < 0.05; Supplementary Figure S4). Furthermore, decreased GGA2 corresponded with poorer recurrence-free survival metrics in six clinical subgroups (BRCA (*p* = 0.0046), HNSC (*p* = 0.034), OV (*p* = 0.016), PAAD (*p* = 0.03), Pheochromocytoma and Paraganglioma (PCPG) (*p* = 0.029), TGCT (*p* = 0.02), reinforcing its potential as a pan-cancer prognostic biomarker (*p* < 0.05; Supplementary Figure S6B). This multi-dimensional analysis underscores GGA2’s regulatory significance in malignant progression and patient outcomes. 

### 2.4. Genomic Alterations of the GGA2 Gene and Their Implications in Cancer

**Figure 5:**
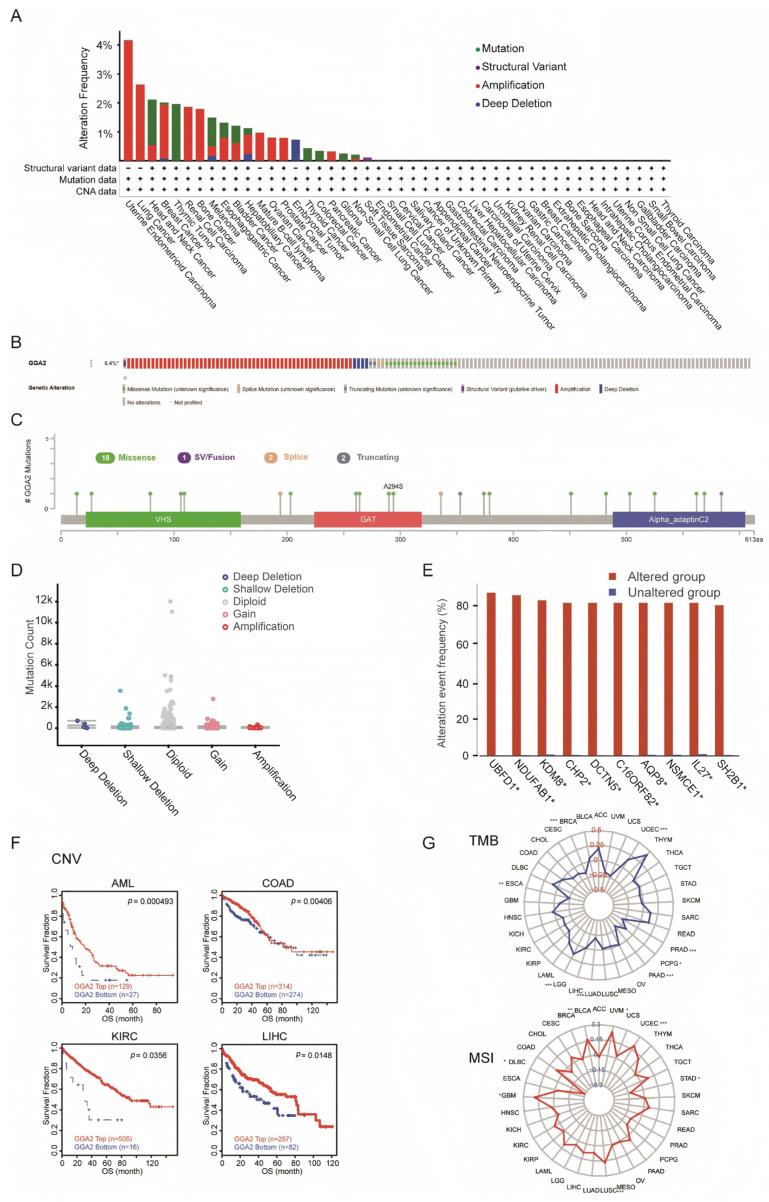
The genetic alterations of GGA2. (**A**) A comprehensive summary of the genetic alterations of GGA2 within the TCGA datasets is provided. (**B**) A detailed summary of the structural variants, mutations, and copy-number alterations of GGA2. (**C**) The mutation types, the quantity of mutations, and the precise sites of the GGA2 genetic alterations are meticulously detailed. (**D**) The types of alterations that GGA2 undergoes in pan-cancer are systematically presented. (**E**) The alteration frequency of related genes in both the GGA2-altered group and the unaltered group is clearly shown. (**F**) Kaplan–Meier plots were generated using the TIDE web-based tool to elucidate the prognostic significance of GGA2 copy-number variations (CNVs) in four specific cancer types. (**G**) Radar charts are employed to vividly depict the association between tumor mutational burden (TMB, presented at the top), microsatellite instability (MSI, presented at the bottom), and GGA2 in pan-cancer. In these radar charts, the dashed-line circle represents a correlation coefficient of 0. Solid-line intersections (either red or blue) within the dashed-line circle signify negative correlation coefficients, indicating an inverse relationship, while those outside the circle denote positive coefficients, suggesting a direct relationship. Statistical significance is denoted as follows: * *p* < 0.05, ** *p* < 0.01, and *** *p* < 0.001, which helps in objectively evaluating the strength and significance of these associations.

We explored genomic alterations of the GGA2 gene by examining copy number variations (CNV) and single nucleotide variations (SNV) to assess their potential role in carcinogenesis. Significant GGA2 amplification was noted in uterine endometrial carcinoma (UEC), and a considerable mutation frequency (>2%) was detected in head and neck squamous cell carcinoma (HNSC) (Figure 5A). Amplifications emerged as the most common type of genetic modification in GGA2, along with the identification of missense mutations and deep deletions (Figure 5B). Details regarding the types, locations, and prevalence of these genetic modifications are illustrated in Figure 5C. Among these, the A294S missense mutation in GGA2 was particularly notable, predominantly occurring in GAT. The most frequently observed copy-number alterations in GGA2 involved gains and maintenance of diploidy (Figure 5D). We also examined genetic changes in additional genes such as UBFD1 (*p* < 0.05), NDUFAB1 (*p* < 0.05), KDM8 (*p* < 0.05), CHP2 (*p* < 0.05), DCTN5 (*p* < 0.05), C16ORF82 (*p* < 0.05), AQP8 (*p* < 0.05), NSMCE1 (*p* < 0.05), IL27 (*p* < 0.05) and SH2B1 (*p* < 0.05), finding elevated alteration rates in these genes among the GGA2-altered group (Figure 5E). Survival analyses indicated that lower levels of GGA2 CNV were associated with worse survival outcomes in acute myeloid leukemia (AML, *p* = 0.000493), COAD (*p* = 0.00406), KIRC (*p* = 0.0356), and LIHC (*p* = 0.0148) (Figure 5F). Additionally, we assessed the correlation between GGA2 expression, tumor mutational burden (TMB), and microsatellite instability (MSI) across various cancers. A positive correlation was observed in LGG (*p* < 0.001) and UCEC (*p* < 0.001) between GGA2 expression and TMB, while a negative correlation was noted in BRCA, esophageal carcinoma (ESCA, *p* < 0.01), LUAD (*p* < 0.001), PAAD (*p* < 0.001), PCPG (*p* < 0.05), and PRAD (*p* < 0.001). Regarding MSI, GGA2 expression showed a negative correlation in DLBC (*p* < 0.05) and STAD (*p* < 0.05), but a positive correlation in BLCA (*p* < 0.01), GBM (*p* < 0.05), LUSC (*p* < 0.001), UCEC (*p* < 0.001), and UVM (*p* < 0.05) (Figure 5G).

### 2.5. Relationship Between GGA2 Expression, DNA Methylation, and CTL Infiltration

**Figure 6:**
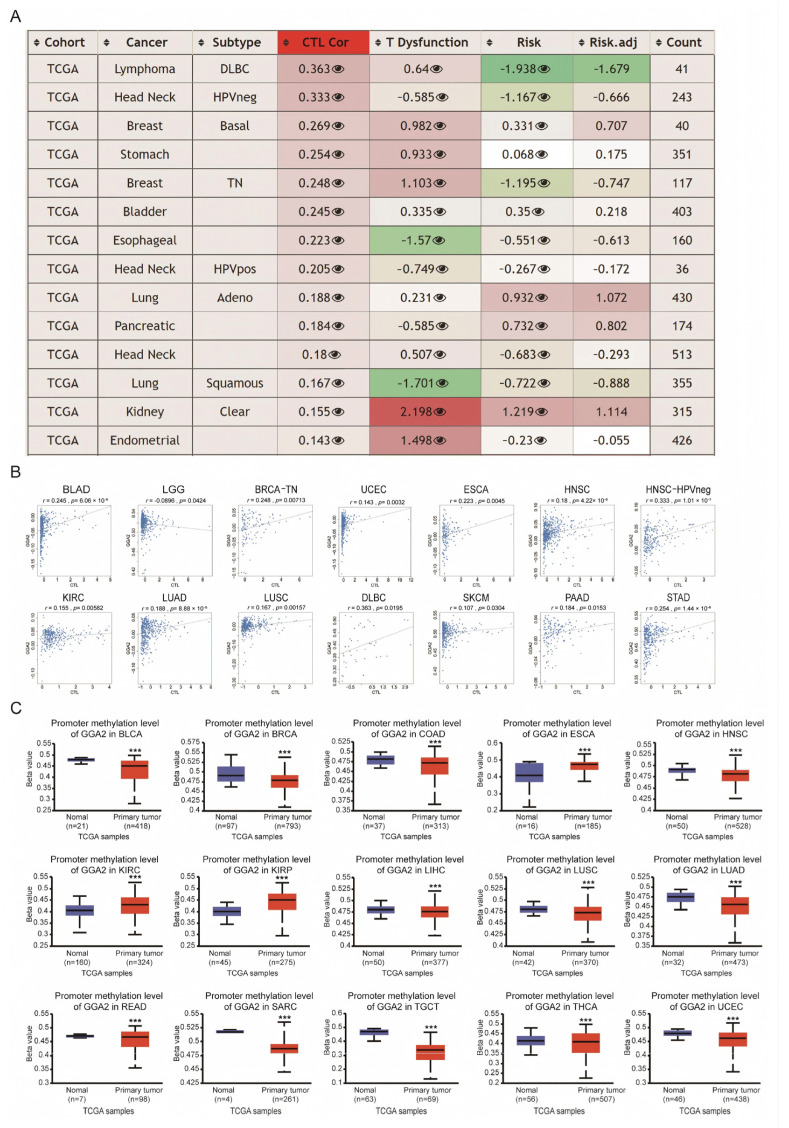
GGA2’s involvement in cancer epigenetic modulations. (**A**) The table demonstrating the correlations between the methylation level of GGA2 and CTL-related factors was obtained from the methylation module of the TIDE web tool. In this table, the third column represents the correlation with CTL, while the fourth column pertains to the z-score of CTL dysfunction for the interaction term. (**B**) The associations between GGA2 methylation levels and CTL markers, along with the survival analyses categorized by high- and low-methylation of GGA2, are presented in the scatter plot. (**C**) The promoter methylation level of GGA2 in various cancers is also examined, *** *p* < 0.001.

Our study delved into the relationship between methylation of the GGA2 promoter, cytotoxic T lymphocyte (CTL) infiltration, and the risk of different cancer subtypes utilizing the TIDE database. The 14 leading cancer subtypes associated with CTL are highlighted in Figure 6A. Scatter plots examining these subtypes indicated a link between CTL infiltration and methylation of the GGA2 promoter (Figure 6B). Additionally, by employing the UALCAN platform, we analyzed the GGA2 promoter methylation levels in both patient and control groups across a spectrum of cancers. Elevated methylation levels in the GGA2 promoter were observed in 15 types of cancer including BLCA, BRCA, COAD, ESCA, HNSC, KIRC, KIRP, LIHC, LUAD, LUSC, rectum adenocarcinoma (READ), sarcoma (SARC), TGCT, THCA, and UCEC (Figure 6C). The promoter methylation level of GGA2 in cancers such as CHOL, CESC, GBM, PAAD, PRAD, PCPG, STAD, and THYM shows no significant difference compared with normal tissues (Supplementary Figure S7).

### 2.6. Impact of GGA2 Alternative Splicing on Cancer Prognosis

**Figure 7:**
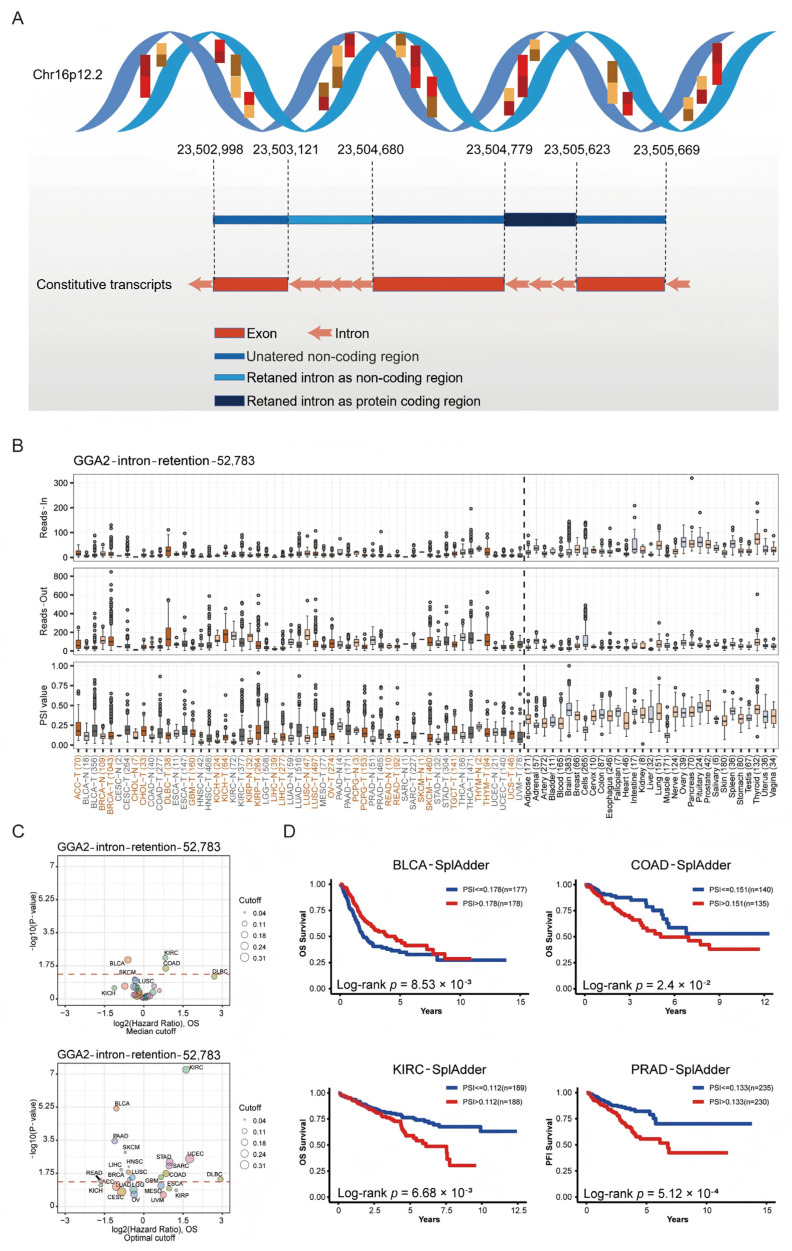
GGA2 alternative splicing correlated to patient prognosis. (**A**) A schematic diagram is provided to elucidate the GGA2 alternative splicing event, Intro_Retention_52783. (**B**) The Reads-in, Reads-out, and PSI values of GGA2_Intro_Retention_52783 are presented for pan-cancer tissues, adjacent tissues, and normal tissues. Here, distinct cancers and their corresponding adjacent tissues are denoted by colorful labels, while non-tumor tissues are represented by black labels. (**C**) A comparison of PSI values is made between tumor and adjacent normal tissues (presented at the top) and between tumor and GTEx normal tissues (presented at the bottom). The red dashed line represents a false discovery rate (FDR) of 0.05. The size of the dots corresponds to the tumor PSI value, and each cancer type is color-coded. (**D**) Kaplan–Meier curves of patients’ OS prediction are plotted. All the data used in this analysis were sourced from the OncoSplicing online web tool. The red and blue lines represent different PSI (Percent Spliced In) values respectively. Blue line: Low-PSI group (patients with a low proportion of splicing events); Red line: High-PSI group (patients with a high proportion of splicing events).

The role of alternative splicing in cancer development is well-documented. Using the Oncosplicing platform, we identified various GGA2 splicing events across cancers. Figure 7A illustrates the Intron_Retention_52783 event, detailing its splice site and patterns across different cancers. Figure 7B displays the Percent Spliced In (PSI) values, along with Reads-out and Reads-in data, for this splicing event in cancerous and normal tissues. Notably, the PSI values for CHOL, KIRP, LUSC, and rectum adenocarcinoma (READ) were higher compared to normal tissues. Figure 7C outlines the Median and Optimal cutoff values for survival based on the expression of Intron_Retention_52783 in various cancers, showing significant disparities in KIRC and BLCA. Kaplan–Meier survival curves in Figure 7D correlate elevated PSI values in COAD (*p* = 2.4 × 10^−2^) and KIRC (*p* = 6.68 × 10^−3^) with lower overall survival (OS), while increased PSI in PRAD (*p* = 5.12 × 10^−4^) corresponds with a decrease in progression-free interval (PFI). These observations highlight the significant association of GGA2 alternative splicing with cancer prognosis.

### 2.7. GGA2 Interactions in Cancer: From DNA Repair to Immune Regulation

**Figure 8:**
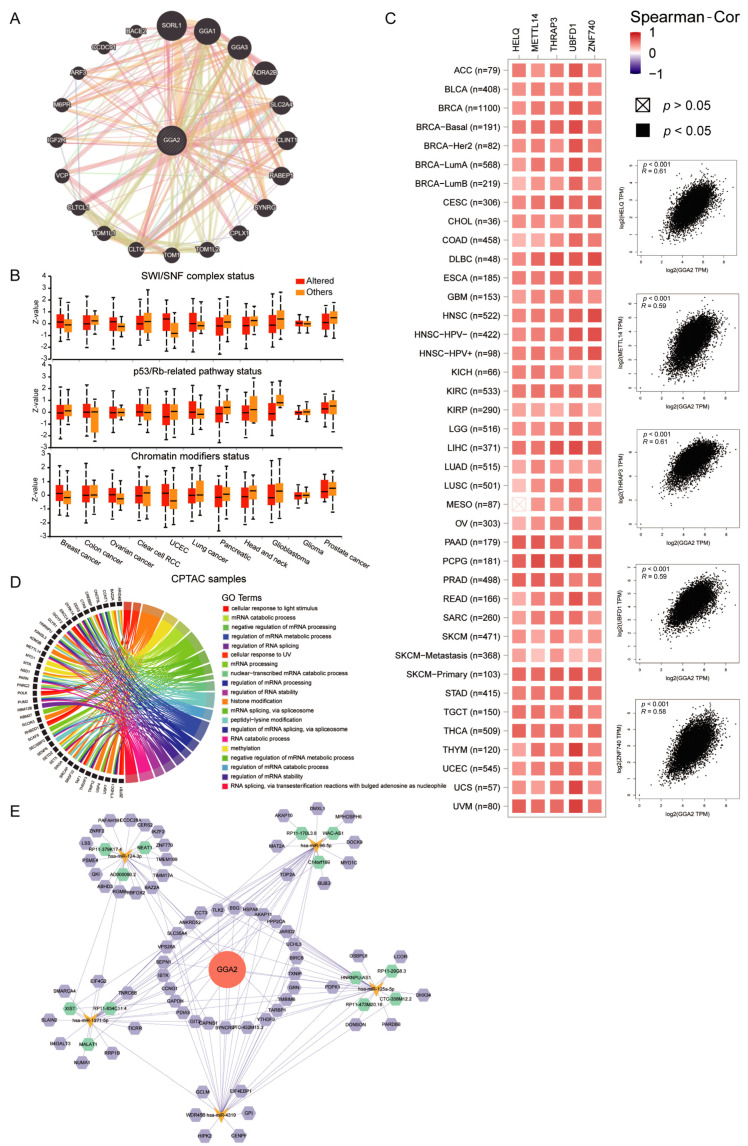
(**A**) The gene–gene interaction network of GGA2 was sourced from GeneMANIA. This network offers a visual and comprehensive illustration of the relationships between GGA2 and other genes within the cellular genetic landscape. (**B**) Using the UALCAN web-based tool, box plots were generated to contrast the expression of GGA2 between pathway-level somatically altered and non-altered groups across 11 distinct cancer types. (**C**) On the GEPIA2.0 platform, a detailed correlation analysis was carried out between GGA2 and its top 5 co-expressed genes. This analysis was executed both for each individual cancer type (**left**) and for the collective pool of all cancer samples (**right**). The term “Partial_Cor” refers to partial correlation. (**D**) Circle plots were constructed to display the Gene Ontology (GO) pathways enriched by the top 100 GGA2 co-expressed genes identified on GEPIA2.0. In these plots, only the top gene of each pathway is listed at the left-hand end of the corresponding color-coded band. (**E**) CeRNA networks of GGA2 were meticulously constructed. In these networks, the red circle represents the hub gene, GGA2. The yellow one symbolizes the miRNAs, the green hexagons denote the lncRNAs, and the purple hexagons represent the circRNAs.

We employed GeneMANIA to build a gene–gene interaction network to probe the interconnections and co-expression patterns of GGA2 in cancer. This study pinpointed the top 20 genes that frequently interact with GGA2, with Midkine (MDK) emerging as the most strongly correlated (Figure 8A). Analysis through UALCAN showed that expression levels of GGA2 were significantly reduced in cancers with alterations in the p53/Rb pathway, the SWI/SNF (Switch/Sucrose Non-Fermentable) complex, and chromatin modification, particularly in PAAD, HNSC, GBM, and PRAD (Figure 8B). Utilizing GEPIA2.0, we identified key genes such as HELQ (R = 0.61), MELLTL14 (R = 0.59), THRAP3 (R = 0.61), UBFD1 (R = 0.59), and ZNF740 (R = 0.58) that were consistently co-expressed with GGA2 across various cancer types (Figure 8C). Gene Ontology (GO) enrichment analysis revealed GGA2’s involvement in crucial cellular functions including cytoskeletal organization and ciliary transport mechanisms (Figure 8D). Furthermore, pathway analysis via GSEA of KEGG pathways underscored the significant linkage of GGA2 with extracellular matrix (ECM) receptor interactions, neuroactive ligand–receptor interactions, and immune system pathways (Supplementary Figure S5).

Our investigation into microRNA (miRNA) interactions through databases like StarBase predicted eight target miRNAs of GGA2 influencing circRNAs and lncRNAs, uncovering 69 target circRNAs and 13 target lncRNAs. These discoveries facilitate the development of ceRNA networks, providing insights into possible therapeutic targets related to GGA2 (Figure 8E). Collectively, these findings illustrate the multifaceted roles of GGA2 in cancer biology, particularly in tumor suppression within prostate cancer.

### 2.8. Analysis of GGA2 Gene Expression and RNA Modification Genes in Various Tumor Stem Cells

**Figure 9:**
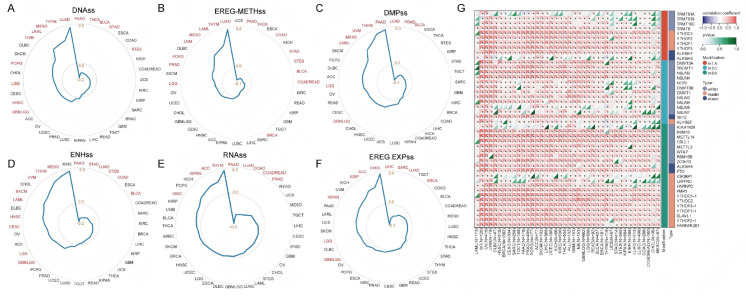
Correlation of GGA2 with tumor stemness and RNA modifications. (**A**–**F**) Radar charts of the correlation between GGA2 and stemness indicators (DNAss, EREG-METHss, DMPss, ENHss, RNAss, EREG-EXPss). Blue lines: correlation coefficients; red marks: significant correlations (*p* < 0.05) in cancer types. (**G**) Heatmap of the correlation between GGA2 and RNA modification genes (m1A, m5C, m6A). Colors indicate the strength of the correlation (red: positive; blue: negative). *p*-values and significance (asterisks) are indicated in the figure.

We analyzed the relationship between GGA2 and the tumor stemness index and found that the association was significantly cancer type-specific. Among various stemness indicators such as DNAss, EREG-METHss, DMPss, ENHss, RNAss, and EREG.EXPss, GGA2 was significantly positively correlated with these indicators in tumors such as GBMLGG, LGG, and KIPAN, suggesting that it may be associated with the maintenance of stemness characteristics of tumor cells and possibly linked to enhanced invasion and drug resistance. Conversely, in tumors such as ESCA, COAD, and KICH, GGA2 was significantly negatively correlated with stemness indicators (Figure 9A–F). Further studies revealed that GGA2 expression was related to the methylation status of genes, and tumors with high GGA2 expression exhibited a stem cell-like RNA expression profile. Additionally, in most tumor types, GGA2 mRNA expression was significantly positively correlated with m1A, m5C, and m6A RNA modification enzymes, except in THYM where it was negatively correlated (Figure 9G). These results suggest that RNA modifications may be involved in the tumor progression mechanism related to GGA2. This study indicates that GGA2 can serve as a potential biomarker for high stemness characteristics of tumors and provides a new target for treating related invasive phenotypes.

### 2.9. Role of GGA2 in Immune Regulation and Cancer Infiltration

**Figure 10:**
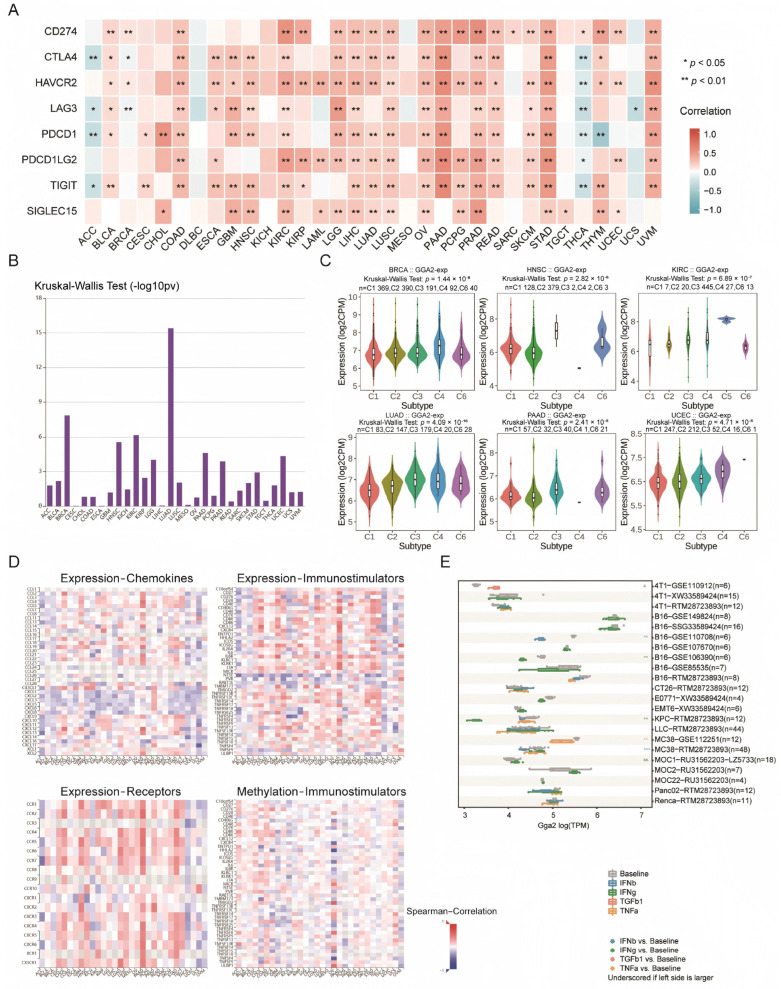
GGA2 was reversely correlated to immune infiltration and cytokine interactions. (**A**) A heatmap was generated to illustrate the associations between immune checkpoints and GGA2 expression across pan-cancer. (**B**) The correlations between GGA2 and immune subtypes were obtained from the TSIDB online tool. (**C**) The expression of GGA2 in six immune subtypes across six different cancer types was examined. (**D**) Heatmaps were presented to show the correlations between GGA2 expression and chemokines (**top-left**), receptors (**bottom-left**), and immunostimulators (**top-right**). Additionally, heatmaps depicting the correlations between GGA2 promoter methylation levels and immunostimulators (**bottom-right**) were included. (**E**) Multiple box plots were retrieved from the TISMO web tool to represent the GGA2 expression in cancer cell lines before and after cytokine treatment. *, **, *** represent *p* < 0.05, *p* < 0.01, and *p* < 0.001 respectively.

Our comprehensive co-expression analysis across 33 cancer types investigated the immunological influence of GGA2. A heatmap demonstrated that GGA2 was co-expressed with the majority of immune checkpoint genes across various cancers, with notable exceptions including CESC, DLBC, KICH, mesothelioma (MESO), sarcoma (SARC), TGCT, and UCS (*p* < 0.05) (Figure 10A). In 25 of these cancers, the strongest positive correlations were observed with HAVCR2, TIGIT, and CD274, particularly in KIRC, OV, LIHC, LUSC, PRAD, and STAD, underscoring GGA2’s potential involvement in immune checkpoint regulation. Using TISIDB, we explored GGA2’s expression across various immune subtypes in cancers, identifying significant associations in nine cancer types. The most notable correlations are summarized in a histogram and detailed further in Figure 10C. Additional examinations revealed that GGA2 generally exhibited a negative correlation with immunostimulators, chemokines, and their receptors across cancers, as shown in heatmaps (Figure 10D).

Lastly, the TISMO web tool was employed to evaluate the expression levels of GGA2 in cancer cell lines both prior to and following cytokine treatment. Post-treatment findings indicated a reduction in GGA2 expression following exposure to IFN-g in three cell lines; similar decreases were also noted after IFN-b treatment in two cell lines, and following treatment with TNF-b1 and TNF-a in one cell line each (Figure 10E). These findings highlight GGA2’s significant association with an immunosuppressive milieu in several cancers, which may be related to its interactions with immunostimulators and checkpoint regulation. This multidimensional analysis provides critical insights into the complex mechanisms by which GGA2 is associated with immune modulation and cancer progression.

### 2.10. GGA2 and M2 Macrophage Infiltration in Cancer Immunity

**Figure 11:**
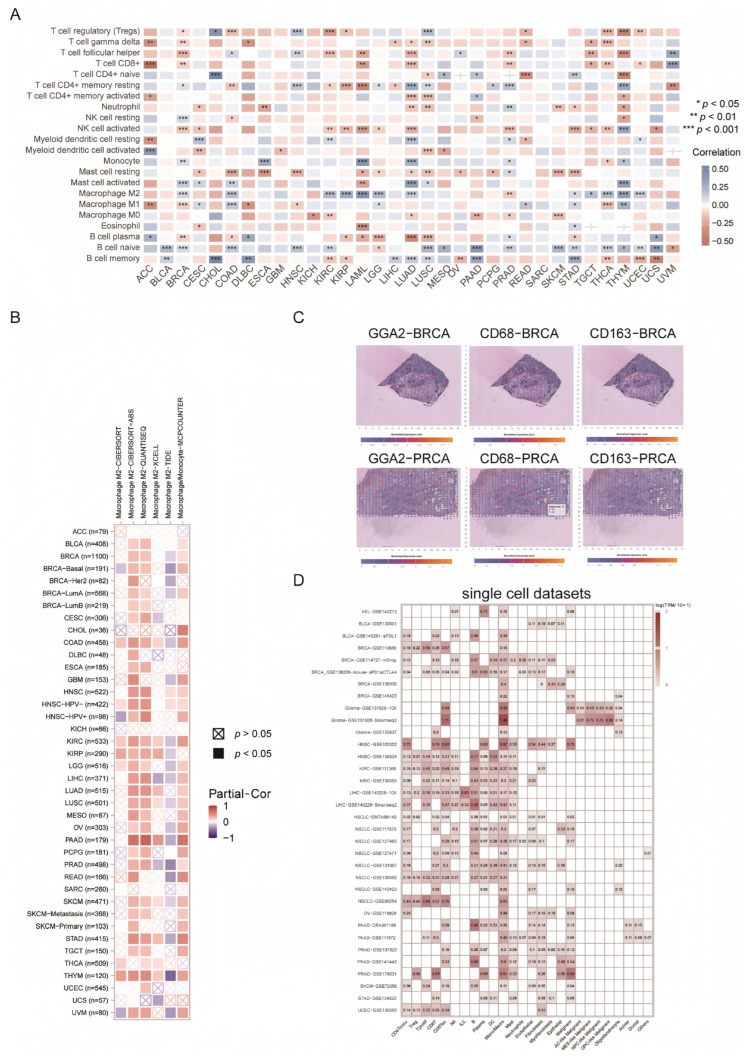
GGA2 has been identified as a biomarker for M2 macrophage infiltration across various cancer types in pan-cancer studies. (**A**) The CIBERSORT algorithm was utilized to comprehensively calculate the immunocyte infiltration patterns within pan-cancer datasets. (**B**) Multiple algorithms integrated within the TIMER2.0 platform were employed to accurately calculate the extent of M2 macrophage infiltration. “Partiall_Cor” denotes partial correlation, a statistical metric that is instrumental in discerning the relationships between variables. (**C**) Spatial transcription sections were generated to visually represent the spatial expression profiles of GGA2, along with the markers CD68 and CD163. In these sections, the color of the dots corresponds to the expression levels of these markers. (**D**) The expression of GGA2 in cancer single-cell clusters was obtained from the TISCH online tool.

We investigated the association of GGA2 at the immunocyte level to understand its potential as a cancer immunomarker. Using the CIBERSORT algorithm, 22 significant correlations between various immunocytes and GGA2 were established. A key finding was the positive correlation of GGA2 with M2 macrophages across twelve cancer types, including BRCA, COAD, KIRC, KIRP, AML, LGG, LUAD, STAD, TGCT, THCA, THYM, and UCEC. In contrast, GGA2 showed negative correlations with regulatory T cells (Tregs) in seven cancer types (Figure 11A). Utilizing TIMER2.0, we expanded this analysis to include various cancers, finding significant links between M2 macrophage infiltration and GGA2 expression, notably in BLCA, BRCA-lumB subtype, CHOL, DLBC, HNSC, LUAD, mesothelioma (MESO), PCPG, SKCM in both metastatic and primary forms, TGCT, THCA and UCEC (Figure 11B). Spatial transcriptional data from SpatialDB were analyzed for breast and prostate cancers (BRCA and PRCA), revealing a potential co-expression of GGA2 with M2 macrophage markers CD68 and CD163 (Figure 11C). Additionally, single-cell analysis from TISCH suggested the expression of GGA2 in M2 macrophages or malignant cells across nearly all studied cell lines (Figure 11D). These multi-dimensional data indicate a robust association between GGA2 and M2 macrophages, suggesting GGA2 as a potent marker for investigating macrophage roles in cancer.

### 2.11. GGA2 and Its Correlation with Immunomodulation in Cancer

**Figure 12:**
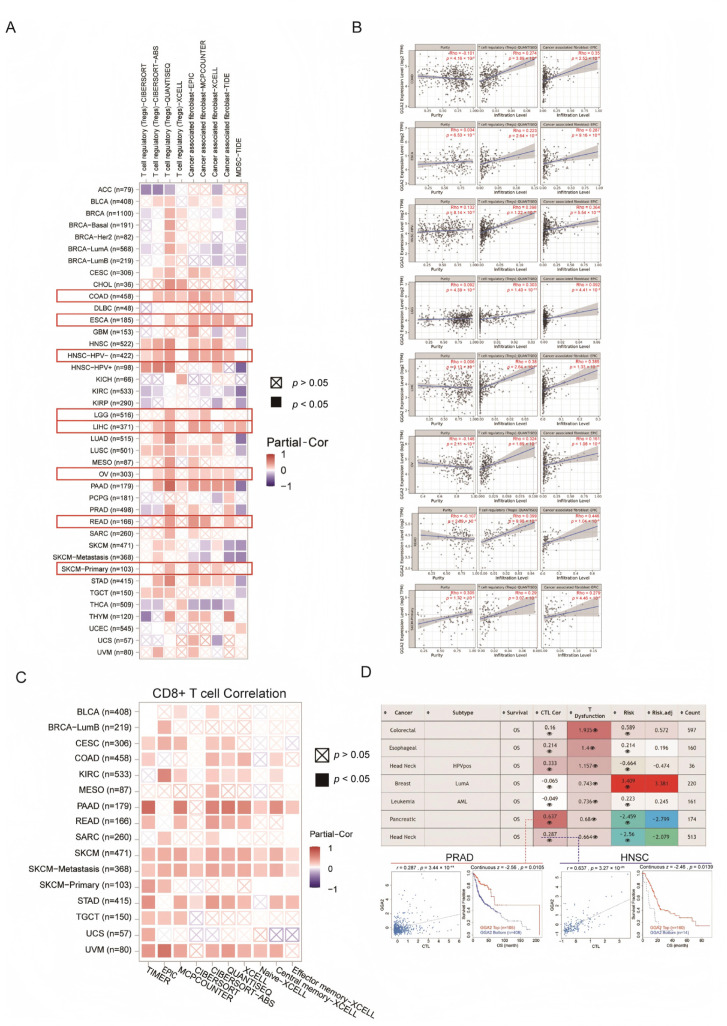
GGA2 was correlated to Tregs, CAFs, MDSC infiltration, and CD8+ T-cell depression. (**A**) A heatmap depicting the associations between the GGA2 level and the infiltration of Tregs, CAFs, and MDSCs was computed using multiple algorithms available on TIMER2.0. The red box within this heatmap serves to emphasize those cancer types where more than two cell types display consistent trends. (**B**) In panel (**A**), scatter plots from TIMER2.0 are utilized to highlight the purity and purity-adjusted correlations between GGA2 and Tregs, CAFs, and MDSCs in eight distinct cancers. (**C**) The correlations between GGA2 and CD8^+^ T-cell infiltration were determined through the use of multiple algorithms. (**D**) The table presents correlations between GGA2 expression and CTL, CTL dysfunction, and risks.

Our analysis extended to other components of the immunosuppressive network. Utilizing TIMER2.0, we examined the relationships between GGA2 and immunosuppressive cells including regulatory T cells (Tregs), cancer-associated fibroblasts (CAFs), and myeloid-derived suppressor cells (MDSCs). Notable positive correlations with at least two of these cell types were detected in several cancers, such as COAD (Tregs: *p* = 3.89 × 10^−6^, CAFs: *p* = 2.52 × 10^−9^), ESCA (Tregs: *p* = 2.64 × 10^−3^, CAFs: *p* = 9.16 × 10^−5^), HPV-negative head and neck squamous cell carcinoma (HNSC-HPV-) (Tregs: *p* = 1.22 × 10^−16^, CAFs: *p* = 5.54 × 10^−14^), LGG (Tregs: *p* = 1.40 × 10^−11^, CAFs: *p* = 4.41 × 10^−2^), LIHC (Tregs: *p* = 2.64 × 10^−13^, CAFs: *p* = 1.33 × 10^−13^), OV (Tregs: *p* = 1.69 × 10^−7^, CAFs: *p* = 1.08 × 10^−2^), rectum adenocarcinoma (READ) (Tregs: *p* = 9.98 × 10^−5^, CAFs: *p* = 1.04 × 10^−5^), and primary skin cutaneous melanoma (SKCM-Primary) (Tregs: *p* = 3.07 × 10^−3^, CAFs: *p* = 4.46 × 10^−3^) (Figure 12A). The most pronounced correlations of GGA2 were with CAFs, and these findings, adjusted for tumor purity, are depicted in Figure 12B. Additionally, the relationship between GGA2 and cytotoxic T lymphocytes (CTLs), key players in immune response suppression, was probed using TIMER2.0. Significant positive correlations were found across various cancers including CESC (*p* < 0.05), COAD (*p* < 0.05), KIRC (*p* < 0.05), PAAD (*p* < 0.05), READ (*p* < 0.05), SARC (*p* < 0.05), SKCM (*p* < 0.05), STAD (*p* < 0.05), TGCT (*p* < 0.05) and UVM (*p* < 0.05) (Figure 12C). Further investigations using the TIDE web tool highlighted a positive association between GGA2 and CTL functionality in HNSC (*p* = 3.44 × 10^−11^) and PAAD (*p* = 3.27 × 10^−21^), revealing an influential link between T-cell dysfunction and GGA2 in these cancers (Figure 12D). This comprehensive assessment suggests GGA2’s involvement in the anti-cancer immune response through interactions with various immunosuppressive cells, highlighting its complex association role in the tumor microenvironment.

### 2.12. Functional Validation of GGA2 in Prostate Cancer

**Figure 13:**
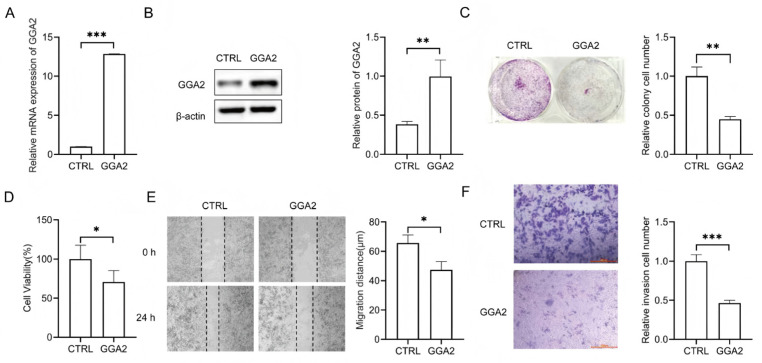
Expression and functional characterization of GGA2 in prostate cancer cells. (**A**) Detection of the mRNA expression levels of GGA2 in the control group (CTRL) and experimental group GGA2-OE. (**B**) Western blot analysis of GGA2 protein detection in CTRL and GGA2-OE. (**C**) Comparative analysis of colony-forming capacity between CTRL and GGA2-OE cell populations. (**D**) The MTT assay demonstrated that GGA2 overexpression inhibited the viability of 22Rv1 cells. (**E**) Temporal analysis of migratory potential demonstrated by wound closure assays in CTRL versus GGA2-OE cellular cohorts at 0, 24 h post-wounding. (**F**) Quantitative assessment of invasive capacity through Transwell assays comparing CTRL and GGA2-OE cells. * *p* < 0.05; ** *p* < 0.01; *** *p* < 0.001.

**Figure 14:**
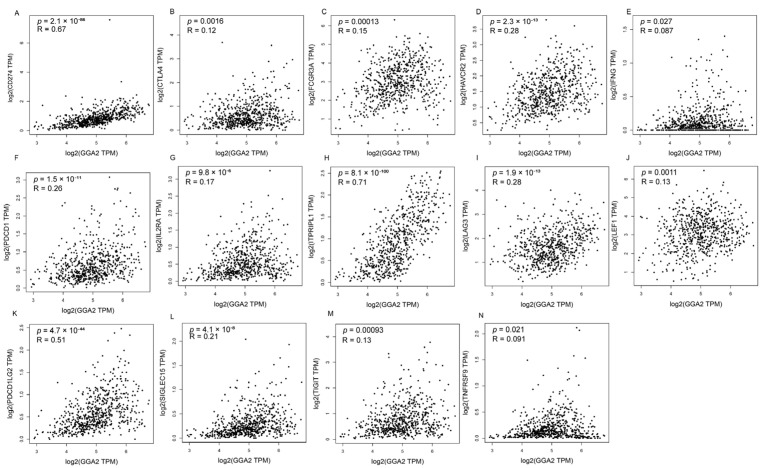
Correlation analysis of GGA2 expression levels with immune checkpoints in PRAD. Relationship between GGA2 and the expression levels of CD274 (**A**), CTLA4 (**B**), FCGR3A (**C**), HAVCR2 (**D**), IFNG (**E**), PDCD1 (**F**), IL2RA (**G**), ITPRPL1 (**H**), LAG3 (**I**), LEF1 (**J**), PDCD1LG2 (**K**), SIGLEC15 (**L**), TIGIT (**M**), TNFRSF9 (**N**).

qRT-PCR analysis revealed significantly higher GGA2 mRNA levels in 22Rv1-GGA2-OE versus CTRL prostate cancer cells (*p* < 0.001; Figure 13A, Supplementary Table S2). Western blot quantification further supported the upregulation of GGA2 protein in the GGA2-overexpressing 22Rv1 cells (*p* < 0.01; Supplementary Table S3). Preliminary functional assays revealed a trend of reduced malignant potential in GGA2-OE cells within this single cell line: compared to CTRL, GGA2-OE cells showed a 55.24% reduction in colony formation (*p* < 0.01; Figure 13C, Supplementary Table S4), a 30% decrease in cell viability (*p* < 0.001; Figure 13D, Supplementary Table S5), an 18.33% slower wound closure rate at 24 h (*p* < 0.05; Figure 13D, Supplementary Table S6), and a 53.57% decrease in Transwell invasion (*p* < 0.001; Figure 13E, Supplementary Table S7). The correlation between immune checkpoints and GGA2 in PRAD was explored using the GEPIA 2 database. The results showed that GGA2 expression was significantly positively correlated with CD274 (*p* = 2.1 × 10^−86^), CTLA4 (*p* = 0.0016), FCGR3A (*p* = 0.00013), HAVCR2 (*p* = 2.3 × 10^−13^), IFNG (*p* = 0.027), IGSF8 (*p* = 6.5 × 10^−17^), IL2RA (*p* = 9.8 × 10^−6^), ITPRPL1 (*p* = 8.1 × 10^−100^), LAG3 (*p* = 1.9 × 10^−13^), LEF1 (*p* = 0.0011), PDCD1 (*p* = 1.5 × 10^−11^), PDCD1LG2 (*p* = 4.7 × 10^−44^), SIGLEC15 (*p* = 4.1 × 10^−8^), TIGIT (*p* = 0.00093), and TNFRSF9 (*p* = 0.021) (Figure 14A–N).

### 2.13. Screening of GGA2-Activating Drugs

**Figure 15:**
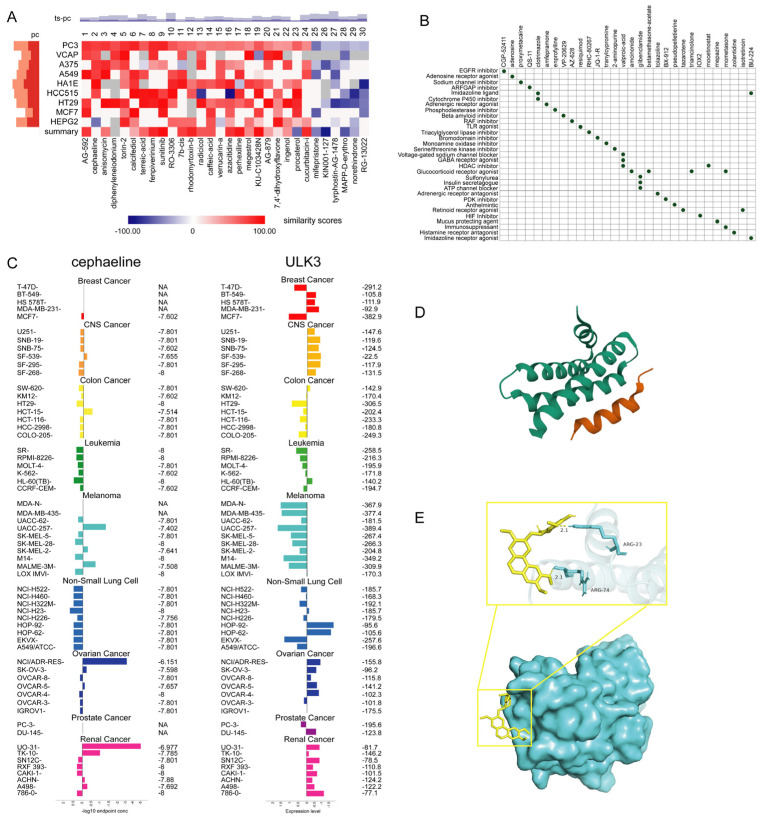
GGA2-activating drug identification, and molecular docking analyses. Evaluation of GGA2 Activation Drug Identification (**A**) Heatmap of the top 30 compounds screened based on the highest upregulated and downregulated genes of GGA2. Similarity scores are indicated by color. pc represents the percentage of total perturbagens, based on the number of rows exceeding a given threshold for all column samples. The height of the deep orange bar indicates a connection percentage ≥ 97.5. ts_pc represents the percentage of total gold standard perturbagens connected to the selected row above the indicated threshold. The height of the deep blue bar indicates a connection percentage ≥ 97.5. (**B**) Scatter plot of MoAs for the top 30 compounds shown in (**A**). (**C**) Evaluation of fulvestrant GI50 values (**left**) and GGA2 levels (**right**) in cell lines. The center line corresponds to the mean −log10(GI50) or GGA2 expression value, each color in the figure represents a type of cancer. (**D**) GGA2 model. (**E**) Image shows the 3D structure of GGA2 for drug binding (blue). Candidate drug 2D structural characteristics (yellow), interacting amino acid residues, molecular forces, and molecular distances are also presented. Abbreviation: LYS, lysine.

It is crucial to identify drugs that can potentially activate GGA2. Using the cMap tool to screen candidate compounds, based on the consistent changes in transcriptional expression in 9 cell lines, we identified 30 compounds predicted to activate GGA2 (Figure 15A). These compounds suggest a potential connection between GGA2 and specific mechanisms within tumor cells (Figure 15B). Further, the COMPARE tool was used to evaluate the GI50 values of these compounds against a series of cancer cell lines, excluding NM-PPI due to the lack of test data. For fulvestrant, the average −log10(GI50) was −4, and in prostate cancer cells, lower GGA2 expression levels were associated with higher GI50 values (Figure 15C). To assess the binding potential of the GGA2 protein with fulvestrant, we conducted a molecular docking study, which indicated that fulvestrant could potentially bind to GGA2 (Figure 15D,E). These results suggest that fulvestrant may be a potential candidate drug for activating GGA2.

## 3. Discussion

The imperative of cancer research in contemporary healthcare cannot be overstated. Cancer arises due to the aberrant expression from transcriptional discrepancies, post-translational changes, or epigenetic alterations in genes, influencing patient outcomes through diverse pathways [12]. Our analysis incorporated gene expression data from 33 cancer-focused datasets via the TCGA and CCLE (Supplementary Table S1), aiming to pinpoint biomarkers for broad-spectrum cancer detection. This study highlights GGA2, a gene newly associated with cancer, elaborating on its clinical relevance, multi-omic characteristics, implications in cancer immunity, and its potential as a pan-cancer therapeutic target. GGA2’s expression was notably diminished in cancers such as PAAD, BRCA, LUSC, LUAD, and THCA, correlating with poorer survival outcomes, whereas its upregulation in THYM was linked to favorable prognosis, illustrating its variable influence depending on the cancer type.

GGA2 expressions were significantly downregulated in cancers such as PAAD, BRCA, LUSC, LUAD, and THCA. Based on survival information from the TCGA database, we observed a consistent correlation between high GGA2 expression and poor prognosis in three pan-cancer types (PCPG, THYM, TGCT). Furthermore, GGA2 expressions in LGG, KIRC, and PAAD were significantly associated with OS, PFI, and DSS. In low-grade glioma (LGG), high GGA2 expression correlated with poor patient prognosis, suggesting a potential tumor-promoting role. GGA2, as a Golgi-associated adaptor protein, is primarily involved in the transport of lysosomal enzymes and the regulation of autophagic flux. Autophagy plays a dual role in tumors, and studies have shown that enhanced autophagy promotes glioma progression [13]. Thus, high GGA2 expressions may facilitate LGG progression by modulating autophagic flux. Aberrant activation of the EGFR signaling pathway is a common driver event in gliomas [14], and elevated GGA2 expression may enhance proliferative signaling by promoting membrane localization or recycling of these receptors. In stark contrast to LGG, TRAF2 promotes cancer progression in clear cell renal cell carcinoma (ccRCC) by inhibiting autophagy [15], while claudin-1 suppresses ccRCC cell proliferation by inducing autophagy [16]. Based on these findings, high GGA2 expression may delay tumor progression in KIRC by supporting autophagic flux. Regarding the differential prognostic value of GGA2 expression across cancers, we hypothesize that modulating GGA2 expression could represent a potent and clinically beneficial strategy tailored to specific tumor types.

Although our analysis revealed associations between GGA2 and survival outcomes in multiple tumors, it can be inferred from the current evidence that the prognostic significance of GGA2 may partly reflect tumor burden, such as GGA2 downregulation in advanced stage tumors, while also potentially functioning as part of a broader molecular network. GGA2 expression may be associated with chromatin modification status, the P53-Rb signaling pathway, among others. P53 is a critical molecule in tumor progression, frequently linked to tumor proliferation, metastasis, and drug resistance [17]. Among the miRNAs regulated by GGA2, MiR-124-3p has been shown to inhibit prostate cancer progression by targeting EZH2 (Enhancer of Zeste Homolog 2) [18], and microRNA-1271-5p suppresses ovarian cancer progression via TIAM1 (T-Cell Lymphoma Invasion and Metastasis 1) and inactivation of the Notch signaling pathway [19]. Therefore, GGA2 may also participate in tumor progression through the regulation of miRNAs.

DNA repair mechanisms play a critical role in maintaining genomic stability, comprising key processes such as homologous recombination deficiency (HRD), mismatch repair (MMR), and non-homologous end joining (NHEJ) [20]. These mechanisms are often activated by chromatin changes during the DNA damage response, halting the cell cycle in the S, G2, and M phases [21]. Our findings propose a link between GGA2 expression and essential genomic stability markers such as tumor mutational burden (TMB) and microsatellite instability (MSI). Significant correlations were observed with TMB in eight cancer types and MSI in seven, underscoring GGA2’s integral role in MMR and HRD systems. Further analysis also revealed its association with critical genomic pathways like those involving p53/RB, SWI/SNF, and chromatin modifiers.

Cellular RNA regulatory mechanisms are intricate, featuring a competitive endogenous RNA (ceRNA) network. miRNAs typically downregulate gene expression by blocking mRNA translation or inducing mRNA degradation. Conversely, circRNAs and lncRNAs can act as miRNA sponges, thus upregulating gene expression by sequestering miRNAs away from their mRNA targets [22]. This complex interplay involves multiple miRNAs and circRNA/lncRNA entities jointly regulating specific target genes, creating an expansive ceRNA network [23]. To elucidate GGA2’s expression dynamics thoroughly, we constructed a detailed ceRNA network that includes non-coding RNAs, circRNAs, and lncRNAs, revealing the gene’s upstream and downstream regulatory interactions in vivo.

GGA2, a molecule with previously undefined functionality, has been revealed for the first time to exhibit correlations with splicing events and cancer cell stemness. These findings offer crucial insights and testable hypotheses for subsequent investigations. Specifically, our study suggests that modulating GGA2-mediated transcriptional splicing may influence the prognosis of certain cancer types, implying a potential role of GGA2 in tumor regulation via RNA splicing pathways. Similarly, the association between GGA2 and various stemness indicators hints at its possible involvement in cancer stem cell characteristics, though this remains a preliminary observation. GGA2 expression is also linked to m1A, m5C, and m6A RNA modification enzymes. Among these, N6-methyladenosine (m6A) RNA modification is recognized as the most prevalent epitranscriptomic modification, regulating processes such as DNA damage repair, downstream adaptive responses (including apoptosis, autophagy, and oncogenic bypass signaling), and cellular stemness [24]. For instance, N^6^-methyladenosine modification of m^6^A RNA methylation and centromere protein K (CENPK)mRNA by Zinc Finger CCCH-Type Containing 13 (ZC3H13) promotes cervical cancer stemness and chemoresistance [25], and the METTL14-miR99a-5p-TRIB2 feedback loop promotes cancer stemness in esophageal squamous cell carcinoma (ESCC) [26]. Additionally, Methyltransferase Like 3 (MLLT3) regulates melanoma stemness and progression by suppressing nuclear translocation of High Mobility Group Box 1 (HMGB1) and Melanoma-Associated Antigen A1 (MAGEA1) m5C modification [27]. Further direct experimental evidence is required to substantiate the relationship between GGA2, RNA splicing, and cancer cell stemness.

Cancer can manipulate non-malignant cells to evade immune detection and may enhance their proliferation. M2-type macrophages are typically associated with tumor progression and play a key role in this process. Targeting these cells to alter their immunosuppressive behavior may help enhance anti-tumor immunity and improve clinical outcomes [28,29]. Our study shows that the level of immune cell infiltration is usually positively correlated with GGA2 expression. Notably, low expression of GGA2 is associated with reduced immune cell infiltration, abnormal function of CD8+ T cells, and increased Tregs, CAFs, and MDSCs, highlighting its broad inhibitory effects. Recent studies have revealed multiple molecular mechanisms underlying the interaction between CAFs and T cell dysfunction. For instance, Wang et al. found that specific knockout of adipocyte enhancer-binding protein 1 (AEBP1) in CAFs enhanced T cell cytotoxicity and inhibited tumor growth, with the mechanism involving AEBP1 binding to cytoskeleton-associated protein 4 (CKAP4) to activate the AKT Serine/Threonine Kinase/Programmed Death-Ligand 1 (AKT/PD-L1) signaling pathway [30]. A disintegrin and mmetalloproteinase 12 (ADAM12) deficiency enhances T cell immune responses and induces tumor rejection in multiple mouse models [31]. In colorectal cancer, a CAF subpopulation (TinCAF) highly expresses nectin cell adhesion molecule 2 (NECTIN2), which directly interacts with T cells to inhibit effector T cell function and promote T cell exhaustion [32]. CAFs can induce apoptosis of antigen-specific CD8+ T cells through PD-L2 and FASL, thereby protecting tumor cells from immune attack [33]. In 21 types of cancer, CD274 (PD-L1) is significantly correlated with GGA2. CD274 is a well-known immune checkpoint protein known to inhibit CD8+ T cell activity and promote M2-type macrophage polarization. For example, Liang et al. demonstrated that PD-L1 in melanoma cells and their secreted extracellular vesicles can induce M2-like macrophage polarization; knockdown of PD-L1 significantly increased the M1/M2 macrophage ratio and enhanced CD8+ T cell activation [34]. In contrast, PD-L1+ macrophages in colorectal cancer exhibit a more M1-like phenotype and are associated with favorable clinical outcomes, suggesting that the relationship between PD-L1 and macrophage polarization may be context-dependent [35]. Our findings suggest that GGA2 may be related to CD274-related immune regulation. Colony formation assays indicated that the overexpression of GGA2 in prostate cancer cells led to a significant decrease in the number of colonies formed, suggesting its function as a growth inhibitor. Furthermore, wound healing assays revealed a reduction in cell migration among GGA2-overexpressing cells, thereby supporting the hypothesis that GGA2 may play a pivotal role in inhibiting metastasis. Additionally, our bioinformatic analyses uncovered a potential novel function of GGA2 in tumor immune modulation. Specifically, GGA2 expression levels exhibited a significant positive correlation with 14 key immune checkpoint molecules. Immune checkpoint molecules (e.g., PD-1, CTLA-4, LAG-3) serve as critical executors of tumor immune evasion; their concerted upregulation typically signifies an immunosuppressive tumor microenvironment [36]. Consequently, GGA2 may not only act as an inhibitor of PRAD cell proliferation, but also extensively modulate the immune checkpoint network through specific mechanisms, thereby reshaping the tumor microenvironment and participating in the process of tumor immune escape. Fulvestrant may exert its therapeutic effect in part by restoring or activating the expression of GGA2, but this requires further experiments to verify the direct regulatory relationship between fulvestrant and GGA2.

The present study has the following limitations: the sample sizes for certain cancer types were relatively small, limiting the statistical power of multivariate models. Multivariate analysis was not included in this study, and therefore it cannot be definitively concluded whether GGA2 is independent of factors such as tumor stage and grade. Future research should employ functional experiments to directly validate its mechanisms in various tumors. Regarding the analyses of alternative splicing and stem cell activity, all findings are correlations derived from public data and computational algorithms, and do not provide evidence of causal relationships. These correlations may be influenced by tumor heterogeneity or other molecular features not considered in the present analysis. Our findings are based on correlation analysis and cannot determine the causal relationship between GGA2 expression and immune characteristics. The observed correlations may result from the direct effect of GGA2, reverse regulation of GGA2 by immune cells, or common upstream regulatory mechanisms. Future research should directly verify its mechanism in various tumors through functional experiments.

## 4. Materials and Methods

### 4.1. Analysis of GGA2 Expression Variability

We sourced standardized datasets of pan-cancer and normal tissues from the University of California, Santa Cruz (UCSC) (https://gtexportal.org/home/; accessed on 8 October 2022), the Cancer Genome Atlas (TCGA) (https://portal.gdc.cancer.gov/; accessed on 9 October 2022), the Therapeutically Applicable Research to Generate Effective Treatments (TARGET) (https://www.target.com; accessed on 9 October 2022) and the Genotype-Tissue Expression (GTEx) (https://gtexportal.org/home/; accessed on 10 October 2022) project. We excluded cancer types represented by fewer than three samples.

We extracted mRNA expression profiles from the TCGA database. Differential expression analyses between tumor and adjacent normal tissues were conducted using log2 transformations and *t*-tests, with a significance threshold set at *p*-value < 0.05. R software (version 4.2.2) facilitated the data analyses, employing the “ggplot2” package for generating box plots. Additionally, GGA2 expression data from cancer tissues were compared to those in the Broad Institute Cancer Cell Line Encyclopedia (CCLE) (https://portals.broadinstitute.org/ccle/; accessed on 12 October 2022). Disparities in GGA2 mRNA levels between cancerous and adjacent normal tissues were further evaluated using the Gene_DE module of TIMER2.0 [37,38] (http://timer.cistrome.org/; accessed on 13 October 2022). Protein expression levels of GGA2, comparing cancerous to normal tissues, were assessed using data from the Clinical Proteomic Tumor Analysis Consortium (CPTAC) via the UALCAN portal [39] (http://ualcan.path.uab.edu/; accessed on 12 October 2026). From the Human Protein Atlas (HPA) (https://www.proteinatlas.org/; accessed on 14 October 2022) [40], immunohistochemical images for 12 tumor types and their corresponding normal tissues were downloaded to assess GGA2 protein expression differences.

### 4.2. Relationship Analysis Between GGA2 Expression and Prognosis

We used the Kaplan–Meier plotter database (http://www.kmplot.com/; accessed on 17 October 2022) [41] to evaluate the impact of GGA2 expression on survival across different cancers. Overall survival (OS), disease-specific survival (DSS), and progression-free interval (PFI) were analyzed as markers to determine the correlation between GGA2 expression levels and patient prognosis. Hazard ratios (HR) with 95% confidence intervals were calculated, and significance was assessed at *p* < 0.05.

### 4.3. Analysis of Cancer-Associated Genomic Alterations and Antigen Correlation of GGA2

The cBioPortal database (http://www.cbioportal.org/; accessed on 18 October 2022) was employed to investigate the genetic alterations of GGA2 in the TCGA pan-cancer datasets [42]. We explored genetic changes and mutation locations using the “Oncoprint,” “Cancer Type Summary,” and “Mutations” modules. The prognostic value of GGA2 copy number variants (CNVs) was analyzed using the Kaplan–Meier curve from the Tumor Immune Dysfunction and Exclusion (TIDE) network tool (cide.ccr.cancer.gov; accessed on 20 October 2022) [43], which also assesses the link between genomic or transcriptional alterations and the efficacy of immunotherapy. Additionally, the “maftools” software package was utilized to compute tumor mutation burden (TMB) and microsatellite instability (MSI), further exploring the associations between TMB, MSI, and GGA2 expression.

### 4.4. Correlation of GGA2 Expression with DNA Methylation

The regulatory impact of GGA2 promoter methylation on its transcriptional activity was investigated through epigenetic profiling. Through the TIDE database’s methylation analysis module [43], correlations emerged between these epigenetic modifications, immune cell dynamics (CTL infiltration), and prognostic indicators in cancer patients. Furthermore, pan-cancer analysis via UALCAN platform uncovered tumor-type-specific methylation signatures in GGA2 regulatory regions.

### 4.5. Clinical Relevance of GGA2 Alternative Splicing

To identify clinically significant alternative splicing (AS) events of GGA2, we accessed the ClinicalAS module of the OncoSplicing server [44] (http://www.oncosplicing.com/; accessed on 20 October 2022). This included searches in the SplAdder and SpliceSeq projects for AS events. We used the PanPlot to illustrate the Percent Spliced In (PSI) values for GGA2 across TCGA cancer types and GTEx normal tissues. The PanDiff chart was applied to contrast the PSI values of AS events, which were identified in more than three cancer types, between cancerous and adjacent normal tissues or GTEx samples. Lastly, a Kaplan–Meier curve was generated to assess the prognostic relevance of these pan-cancerous AS events.

### 4.6. The GGA2 Interaction Network and Analyses of Functional Enrichment

We utilized the GeneMANIA database (http://www.genemania.org; accessed on 3 November 2022) to determine genes with similar expression patterns to GGA2, based on extensive genomics and proteomics data. At the pathway level, we investigated pan-cancer somatic changes and the expression correlations between pathway-related markers and GGA2 on UALCAN and GEPIA2.0 (http://gepia2.cancer-pku.cn/; accessed on 3 November 2022) [45]. Additionally, we identified the top 100 co-expressed genes with GGA2 in pan-cancer using the Similar Gene Detection function of GEPIA2.0. Functional analysis of these genes was conducted using the Gene Ontology (GO) annotations, employing the R packages “clusterProfiler” and “org.Hs.eg.db” with a false discovery rate (FDR) of <0.05.

The expression correlation patterns between GGA2 and its top five co-expressed genes were graphically represented using heatmaps in TIMER2.0 and scatter plots in GEPIA2.0. Subsequent miRNA target prediction integrated five bioinformatic platforms (DIANA-microT, RNA22, miRDB, miRWalk, miRcode), with stringent criteria requiring miRNA identification across ≥3 independent databases to ensure prediction reliability.

StarBase v2.0 provided comprehensive networks for miRNA–lncRNA and miRNA–circRNA interactions (https://starbase.sysu.edu.cn; accessed on 6 November 2022) [46,47], with criteria including mammalian, human, hg19, and stringent CLIP-Data conditions (≥5 clips), with or without Degradome-Data. We utilized Cytoscape (version 3.9.1) to visualize the ceRNA networks, illustrating interactions among noncoding RNAs (ncRNAs), miRNas, and mRNAs.

### 4.7. Cell Culture, Plasmid Construction and Lentiviral Transduction

The human prostate cell line 22Rv1, obtained from the Chinese Academy of Sciences Cell Bank, was cultured in RPMI1640 medium (Shanghai, China) supplemented with 10% FBS (Thermo Fisher Scientific, Waltham, MA, USA). Cells were maintained at 37 °C in a 5% CO_2_ atmosphere. pLVX-Puro-GFP-GGA2 were generated by subcloning GGA2 into pLVX-Puro-GFP vector. Lentiviral particles were packaged in 293T cells using Lipofectamine 3000 (Invitrogen, Carlsbad, CA, USA) following manufacturer’s protocol. Target cells were transduced with either GGA2-OE or control vectors (pLVX-Puro empty vector, Fenghui Biotechnology, Hunan, Changsha, China) for 72 h, followed by selection with 1.5 μg/mL puromycin (Thermo Fisher Scientific, Waltham, MA, USA) for 10 days to establish stable polyclonal populations. 

### 4.8. Quantitative Reverse Transcription PCR (qRT-PCR)

Total RNA from 22Rv1 cells was isolated using TRIzol reagent (Sangon Biotech, Shanghai, China) and reverse-transcribed with PrimeScript RT Master Mix (Takara, Tokyo, Japan). qPCR amplification was conducted using SYBR Green Mix (Takara, Tokyo, Japan) on a Bio-Rad CFX 96 Real-time PCR system with the following cycling parameters: 95 °C/30 s, 40 cycles of 95 °C/15 s, 60 °C/30 s. Primer sequences: GGA2-F: 5′-ATGTCGGAACAGGATTGGTCA-3′; GGA2-R: 5′-GCACCGTTAAGGCATAAAGAGC-3′; GAPDH-F: 5′-GGAAGGTGAAGGTCGGAGTCA-3′; GAPDH-R: 5′-GTCATTGATGGCAACAATATCCACT-3′.

### 4.9. Western Blot Analysis

Protein lysates prepared with RIPA buffer (Beyotime Biotechnology, Jiangsu, Lianyungang, China) containing protease inhibitors were quantified by BCA assay (Beyotime Biotechnology, Jiangsu, Lianyungang, China). SDS-PAGE separated proteins were transferred to PVDF membranes (Millipore, Darmstadt, Hesse, Germany) and probed with anti-GGA2 (1:1000, Bioworld BS6226) and β-actin (1:5000, Bioworld BS6007M) antibodies. Band intensities were quantified using Image J (version 1.5.1) with normalization to β-actin.

### 4.10. Correlation Analysis of Stemness Indicators

This study evaluated the correlation between GGA2 and multiple stemness indicators, including DNAss, EREG-METHss, DMPss, ENHss, RNAss, and EREG-EXPss. The Pearson correlation coefficient (Pearson R) data between each indicator and GGA2 were obtained from the SangerBox online platform (http://sangerbox.com; accessed on 15 September 2025), and radar charts were drawn using MicroBioinfo (http://www.bioinformatics.com.cn; accessed on 28 September 2025) for visualization. Additionally, the Pearson correlation analysis method in SangerBox was used to assess the association between GGA2 and various RNA modifications, and corresponding charts were generated.

### 4.11. CCK8 Proliferation Assay, Colony Formation Assay, Cell Migration Assay

The procedures for the CCK8 proliferation assay, colony formation assay, and cell migration assay were detailed in a prior publication [48].

### 4.12. Examination of GGA2’s Immunological Roles in the Pan-Cancer Microenvironment

In order to explore the potential correlations between immune checkpoint markers and GGA2, we utilized data from a previous study [49]. We downloaded STAR-counts data and corresponding clinical information for 33 types of tumors from the TCGA database. We then extracted data in TPM format and performed normalization using the log2(TPM + 1) transformation. After retaining samples that included both RNAseq data and clinical information for further analysis, in order to conduct precise immunological assessments, we integrated the CIBERSORT algorithm through R package “immunedeconv”. SIGLEC15, TIGIT, CD274, HAVCR2, PDCD1, CTLA4, LAG3, and PDCD1LG2 are transcripts associated with immune checkpoints. We extracted the expression values of these eight genes to observe the expression patterns of genes related to immune checkpoints. Statistical analysis was conducted using R software (version 4.2.2). Results were considered statistically significant when the *p*-value was less than 0.05. In the Subtype module of TISDB (http://cis.hku.hk/TISIDB/; accessed on 10 December 2022), an analysis was conducted to determine the relevance of GGA2 expression to immune subtypes and to ascertain the comparative levels of expression across these subtypes [50]. Heatmaps were constructed to determine the relationships between GGA2 and various biological molecules, including chemokines, chemokine receptors, and immunostimulators, within the designated Immunomodulator modules and Chemokine. In order to assess the impact of cytokine treatment on GGA2 expression, the TISMO web tool (http://tismo.cistrome.org/; accessed on 10 December 2022) was employed to facilitate a comparative analysis of gene expression in the cell lines prior to and following cytokine administration; the inter-group differences were statistically evaluated using DESeq2 through the Wald test.

The TIMER2.0 software was employed for the purpose of analyzing the intercorrelation between M2 macrophage markers and GGA2 through Spearman correlation analysis. Additionally, the spatial expression and overlap of GGA2 with the M2 macrophage markers in breast cancers and prostate cancers were investigated using the SpatialDB (https://www.spatialomics.org/SpatialDB/; accessed on 12 December 2022) [51]. In order to enhance our understanding, we evaluated the expression of GGA2 in different cell subtypes across a range of cancers by utilizing single-cell datasets obtained from the Gene Expression Omnibus (GEO) and the Tumor Immune Single-cell Hub (TISCH) (https://tisch.compbio.cn/; accessed on 12 December 2022) [52]. We used the GEPIA2 database (http://gepia.cancer-pku.cn/; accessed on 12 December 2022) to analyze the correlation between GGA2 and T cell exhaustion markers. The statistical significance was determined by the criteria of *p* ≤ 0.05 and FDR ≤ 0.05.

### 4.13. Immunosuppressive Impact of GGA2 in the Pan-Cancer Environment

We investigated the association between GGA2 expression and the infiltration levels of regulatory T cells (Tregs), myeloid-derived suppressor cells (MDSCs), CD8+ T cells, and cancer-associated fibroblasts (CAFs) with Spearman correlation analysis. Using the TIDE tool, we further explored the effect of GGA2 on T cell dysfunction across multiple cancer subtypes, as well as its correlation with cytotoxic T lymphocyte (CTL)-related prognosis, by Cox proportional hazards (Cox-PH) regression and Pearson correlation analysis. All statistical results were adjusted using two-sided Wald test and two-sided *t*-test, with a *p* value ≤ 0.05 considered statistically significant [53].

### 4.14. Activation Drugs

The cMap “Query” tool was used to screen potential GGA2 activation compounds, using the top 100 upregulated and downregulated genes identified by GGA2 (stratified by the median expression of GGA2). The data were organized and the top 30 compounds were plotted as a heatmap. Scatter plots were drawn using Excel functions to evaluate their mechanism of action (MoA). Then, the expression of GGA2 and the concentration related to 50% growth inhibition (GI50) in various cell lines from the CellMiner database were downloaded. The data were processed using the readxl, impute, and limma packages in R language, and the plots were made using the MicroBioinfo online website. The 3D structures of GGA2 and fulvestrant were obtained from the PDB database and PubChem database, respectively, and docking was performed using AutoDock (version 4.2.6), with the results visualized using PyMOL (version 3.1.0).

### 4.15. Statistical Analyses

All gene expression data, including RNA-seq data derived from the TCGA and GTEx databases, were normalized using the log_2_(TPM + 1) transformation. Batch effects across multiple datasets were corrected using the ComBat algorithm to minimize technical bias. All bioinformatic analyses were implemented in the R software environment. Correlations between variables were assessed using Spearman’s and Pearson’s correlation coefficients. Comparisons between two groups were performed using the two-sided Wald test and two-sided *t*-test. Unless otherwise specified, all results are presented as the mean ± standard deviation (mean ± SD). Non-parametric data were analyzed using the Wilcoxon test, and one-way analysis of variance (one-way ANOVA) was applied for comparisons among three or more parametric groups. For Cox proportional hazards (Cox-PH) regression analysis, hazard ratios (HRs) were consistently reported together with their corresponding 95% confidence intervals (95% CIs) to quantify the association between GGA2 expression and survival outcomes. Multiple testing correction was applied in all statistical analyses, with the false discovery rate (FDR) set as the correction standard and FDR ≤ 0.05 considered statistically significant. All statistical procedures were conducted in RStudio (version 4.2.2), with *p* ≤ 0.05 and FDR ≤ 0.05 indicating statistical significance.

## 5. Conclusions

To conclude, the gene GGA2 serves as a significant prognostic indicator across multiple cancers due to its irregular expression patterns and its connection with adverse outcomes in patients. The dysregulation of GGA2 is associated with several important factors, including mismatch repair (MMR), microsatellite instability (MSI), tumor mutational burden (TMB), and the immune microenvironment of tumors, which impact a variety of cancer types. In light of these observations, GGA2 presents itself as a potentially valuable prognostic marker and therapeutic target, especially in the context of prostate cancer. The targeting of GGA2 may offer considerable therapeutic advantages in cancer treatment. However, further research is necessary to elucidate the precise mechanisms by which GGA2 affects prostate cancer cells.

## Data Availability

The original contributions presented in the study are included in the article/Supplementary Materials. Further inquiries can be directed to the corresponding authors.

## Supplementary Materials

The following supporting information can be downloaded at: https://www.mdpi.com/article/10.3390/ijms27062905/s1.
