# Supplementary material for: Exploring the Role of GGA2 in Cancer Progression: Pan-Cancer Bioinformatics and Experimental Validation in Prostate Cancer
Source: Int J Mol Sci. 2026 Mar 23;27(6):2905. doi: 10.3390/ijms27062905 (PMC13026977; doi:10.3390/ijms27062905)
Supplement: Supplementary file 1 [file ijms-27-02905-s001.zip › Supplementary Figures.pdf]

Supplementary Material

1 Supplementary Figures

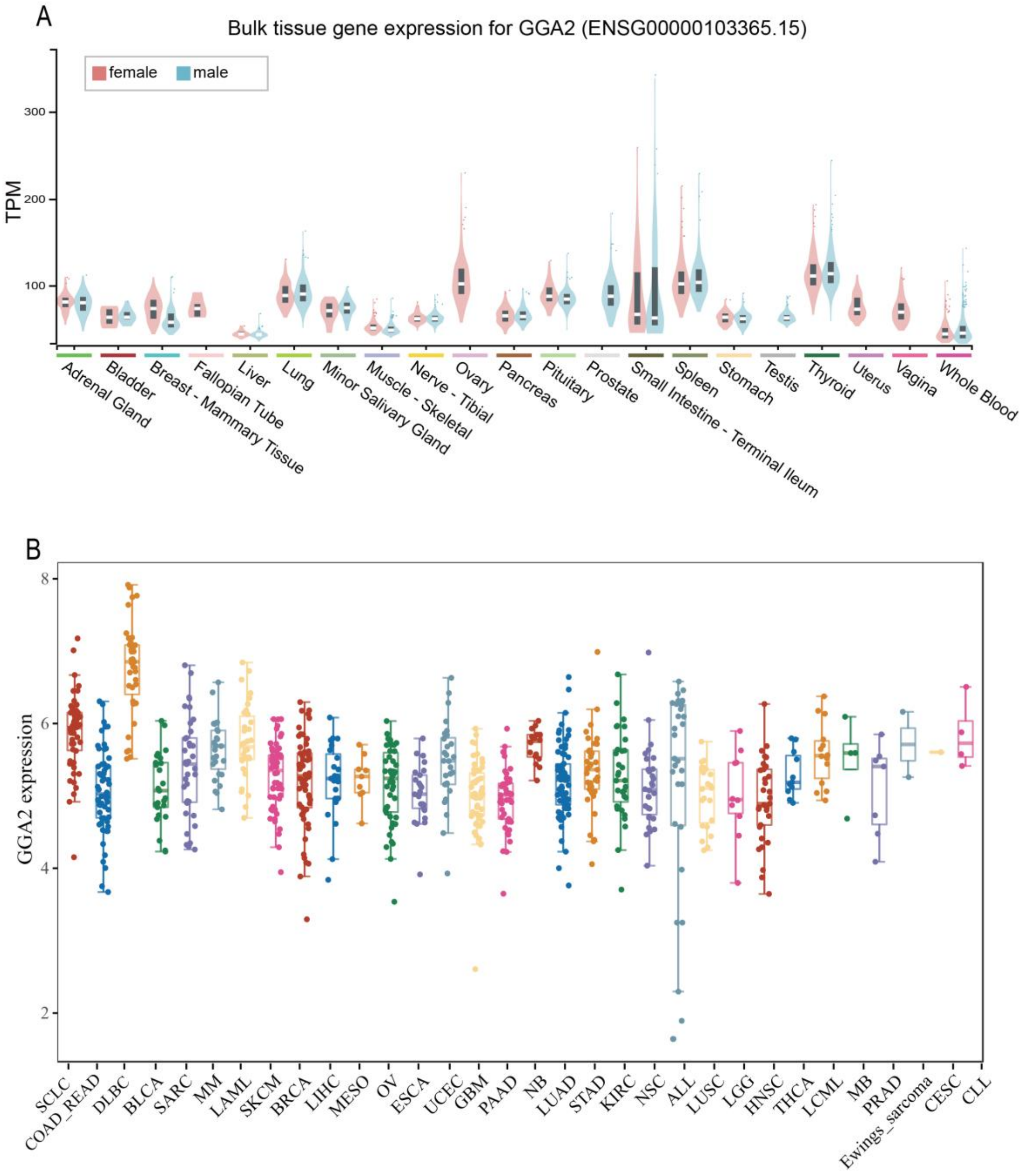

Supplementary Figure S1. Differential expression of GGA2. (A) An in-depth analysis was carried out to determine the expression abundances of GGA2 in a diverse range of male and female tissues. (B) The differential expression of GGA2 mRNA was investigated in nearly all human cell lines present in the CCLE database.

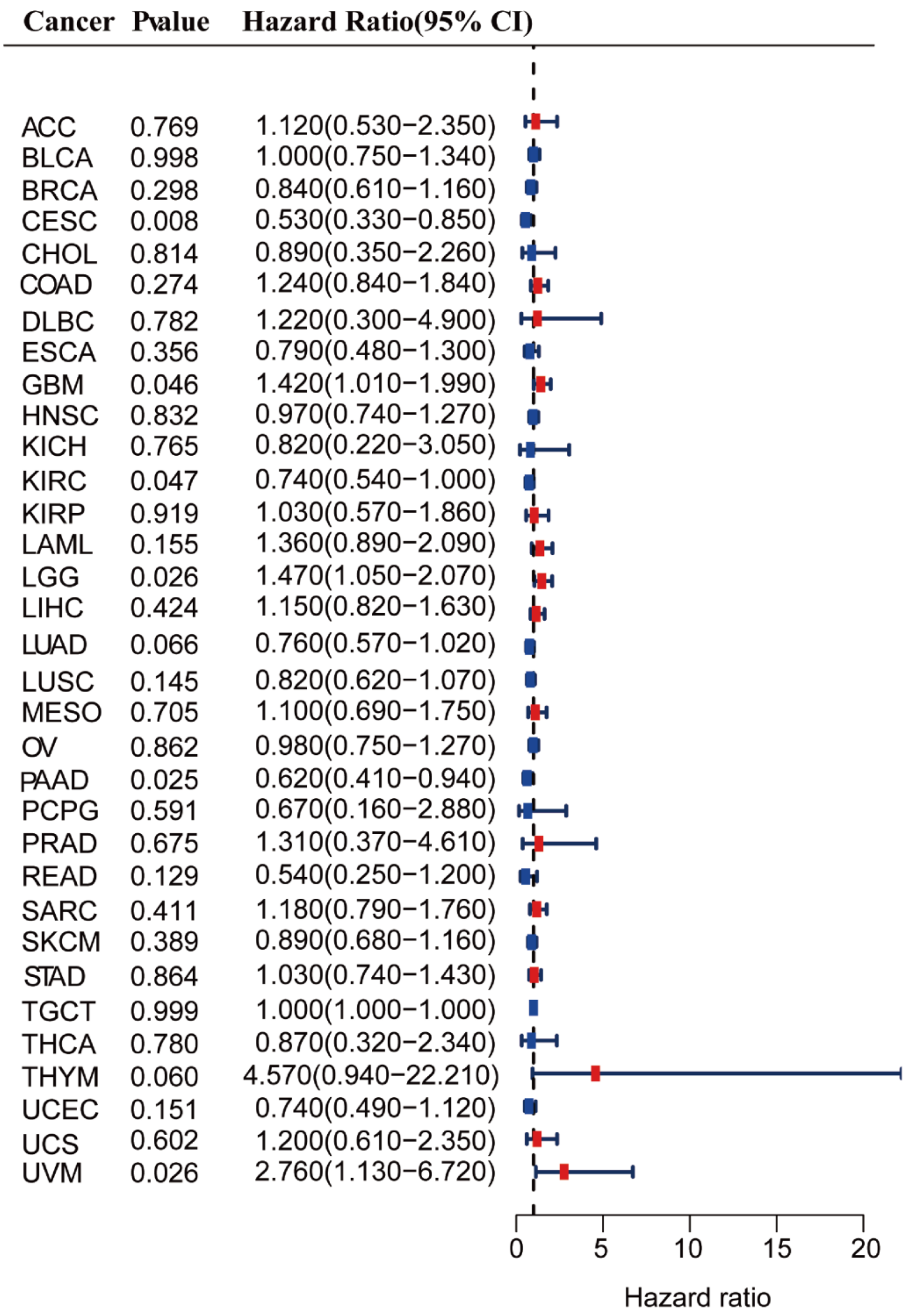

Supplementary Figure S2. The relationship between the GGA2 and overall survival time, measured in days, was examined in both TCGA tumor and non-tumor tissues (Univariate Cox regression analyses). Hazard ratio (HR) value > 1 represents risk factor, whereas HR value < 1 represents favorable factor.

Cancer	Pvalue	Hazard Ratio(95% CI)
ACC	0.588	1.240(0.570−2.680)
BLCA	0.468	0.880(0.620−1.250)
BRCA	0.010	0.560(0.360−0.870)
CESC	0.012	0.500(0.290−0.860)
CHOL	0.767	0.860(0.320−2.300)
COAD	0.460	1.200(0.740−1.970)
DLBC	0.435	0.400(0.040−3.930)
ESCA	0.809	0.930(0.520−1.660)
GBM	0.064	1.410(0.980−2.020)
HNSC	0.260	0.820(0.580−1.160)
KICH	0.745	0.780(0.170−3.490)
KIRC	0.007	0.580(0.400−0.860)
KIRP	0.795	0.910(0.430−1.900)
LAML		NA(NA−NA)
LGG	0.049	1.430(1.000−2.050)
LIHC	0.361	1.230(0.790−1.910)
LUAD	0.418	0.860(0.600−1.240)
LUSC	0.028	0.620(0.400−0.950)
MESO	0.185	1.510(0.820−2.770)
OV	0.809	0.970(0.730−1.280)
PAAD	0.034	0.600(0.380−0.960)
PCPG	0.537	0.580(0.100−3.270)
PRAD	0.848	0.840(0.140−5.180)
READ	0.936	0.960(0.330−2.780)
SARC	0.360	1.230(0.790−1.890)
SKCM	0.751	0.950(0.720−1.270)
STAD	0.159	1.360(0.890−2.070)
TGCT	0.999	1.000(1.000−1.000)
THCA		NA(NA−NA)
THYM	0.219	4.160(0.430−40.600)
UCEC	0.092	0.650(0.390−1.070)
UCS	0.298	1.480(0.710−3.110)
UVM	0.024	2.990(1.160−7.750)

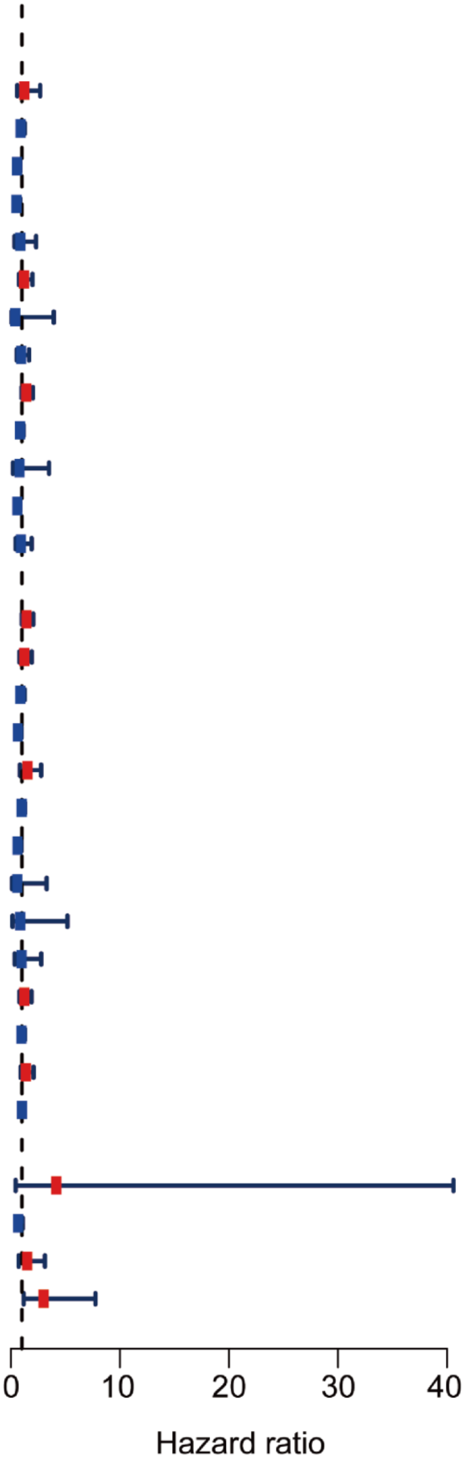

Supplementary Figure S3. The connection between the GGA2 and the DSS was analyzed in TCGA tumor and non-tumor tissues (Univariate Cox regression analyses). Hazard ratio (HR) value > 1 represents risk factor, whereas HR value < 1 represents favorable factor.

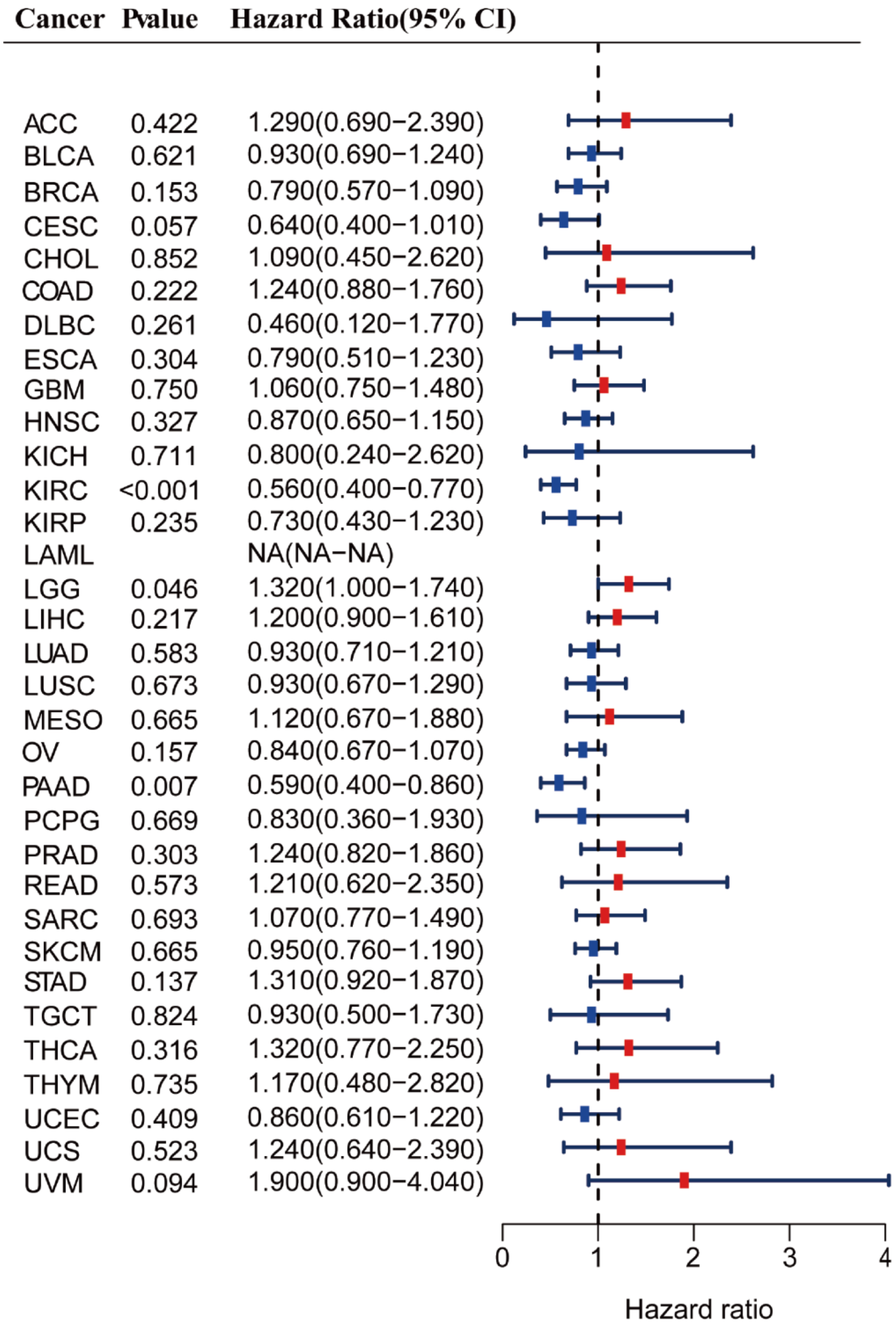

Supplementary Figure S4. The relationship between the GGA2 and the PFI was analyzed in TCGA tumor and non-tumor tissues (Univariate Cox regression analyses). Hazard ratio (HR) value > 1 represents risk factor, whereas HR value < 1 represents favorable factor.

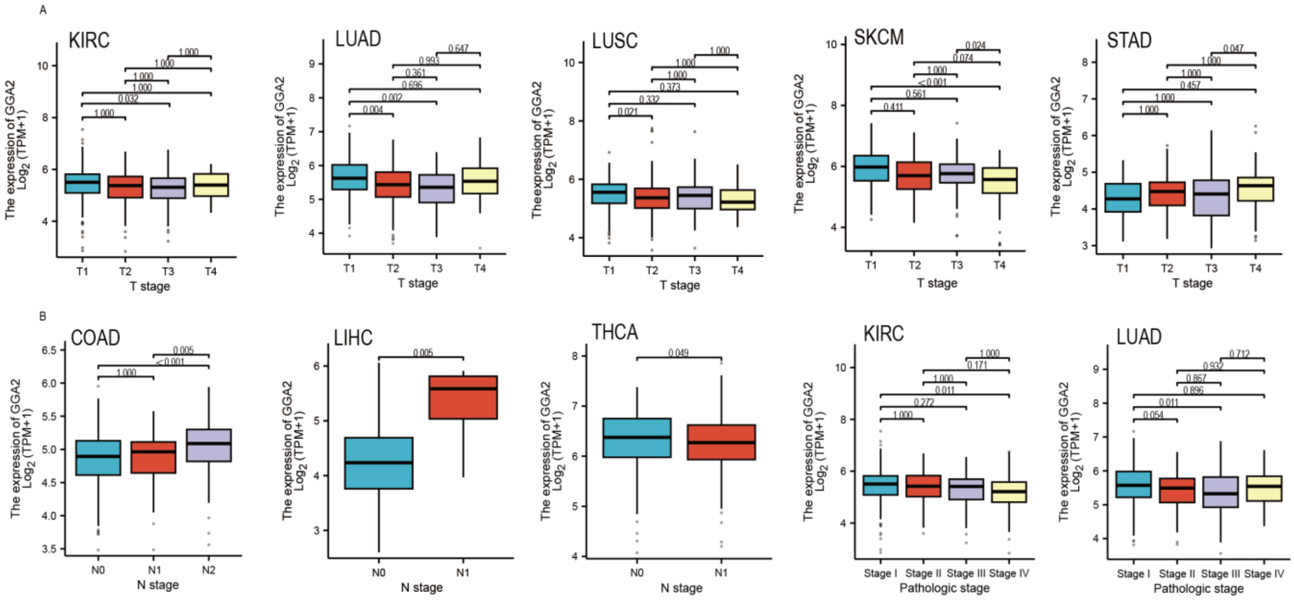

Supplementary Figure S5. An in - depth exploration was carried out to investigate the correlation between the mRNA expression level of GGA2 and the progression of patients' clinicopathological features in the context of pan - cancer.

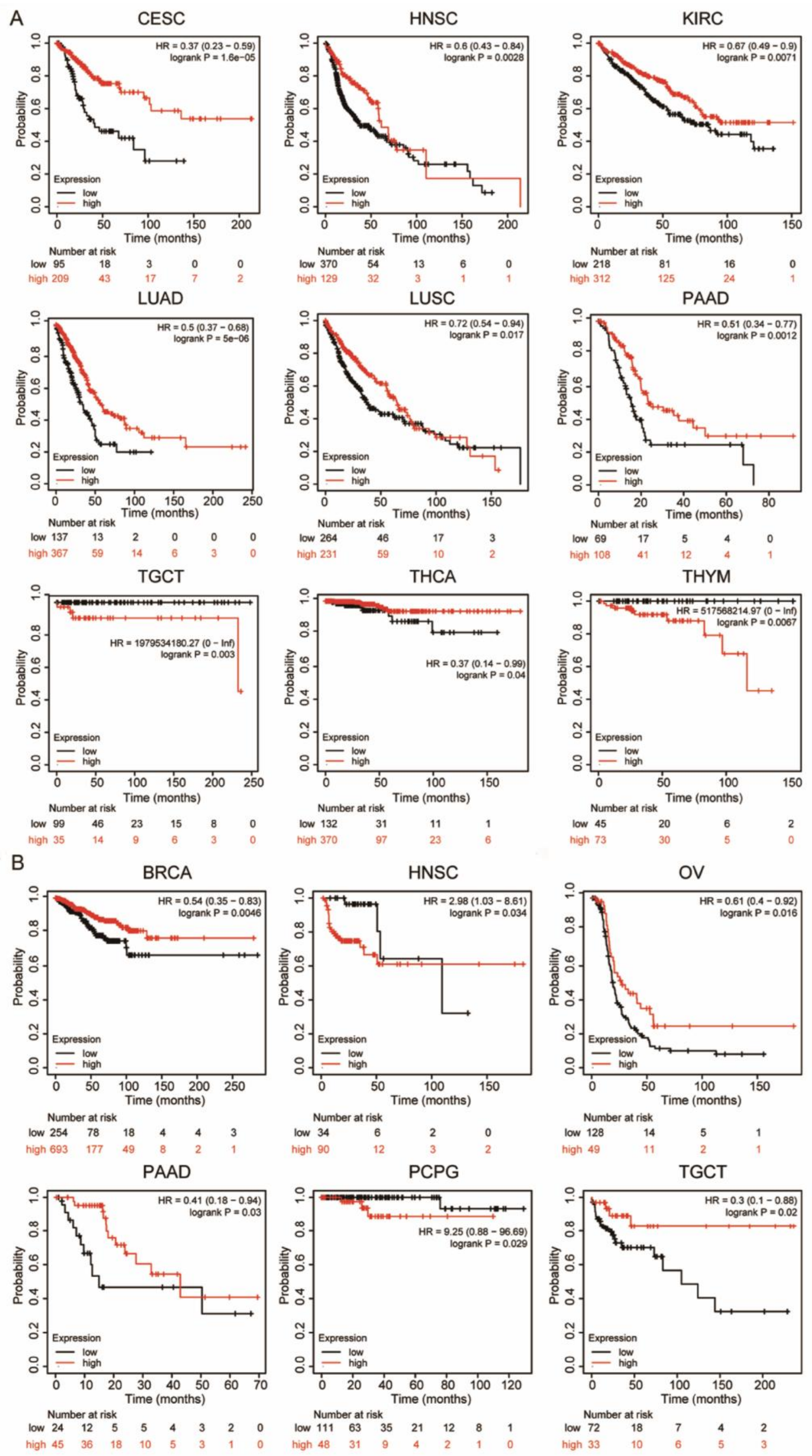

Supplementary Figure S6. A comprehensive Kaplan–Meier analysis was meticulously conducted to investigate the association between the expression of GGA2 and overall survival (OS).

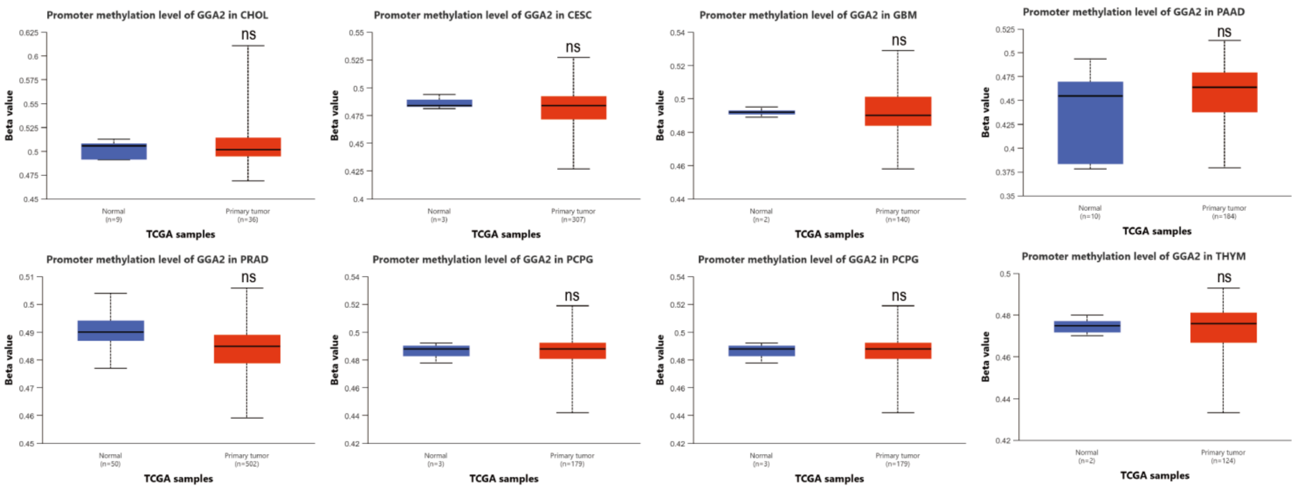

Supplementary Figure S7. GGA2 promoter methylation levels in pan-cancer. The promoter methylation levels of GGA2 in multiple tumors were analyzed based on the UALCAN database. The vertical axis represents the Beta value (0–1 range), indicating the methylation level; the blue boxes represent normal tissues, and the red boxes represent primary tumor tissues. ns, $p>0.05$.

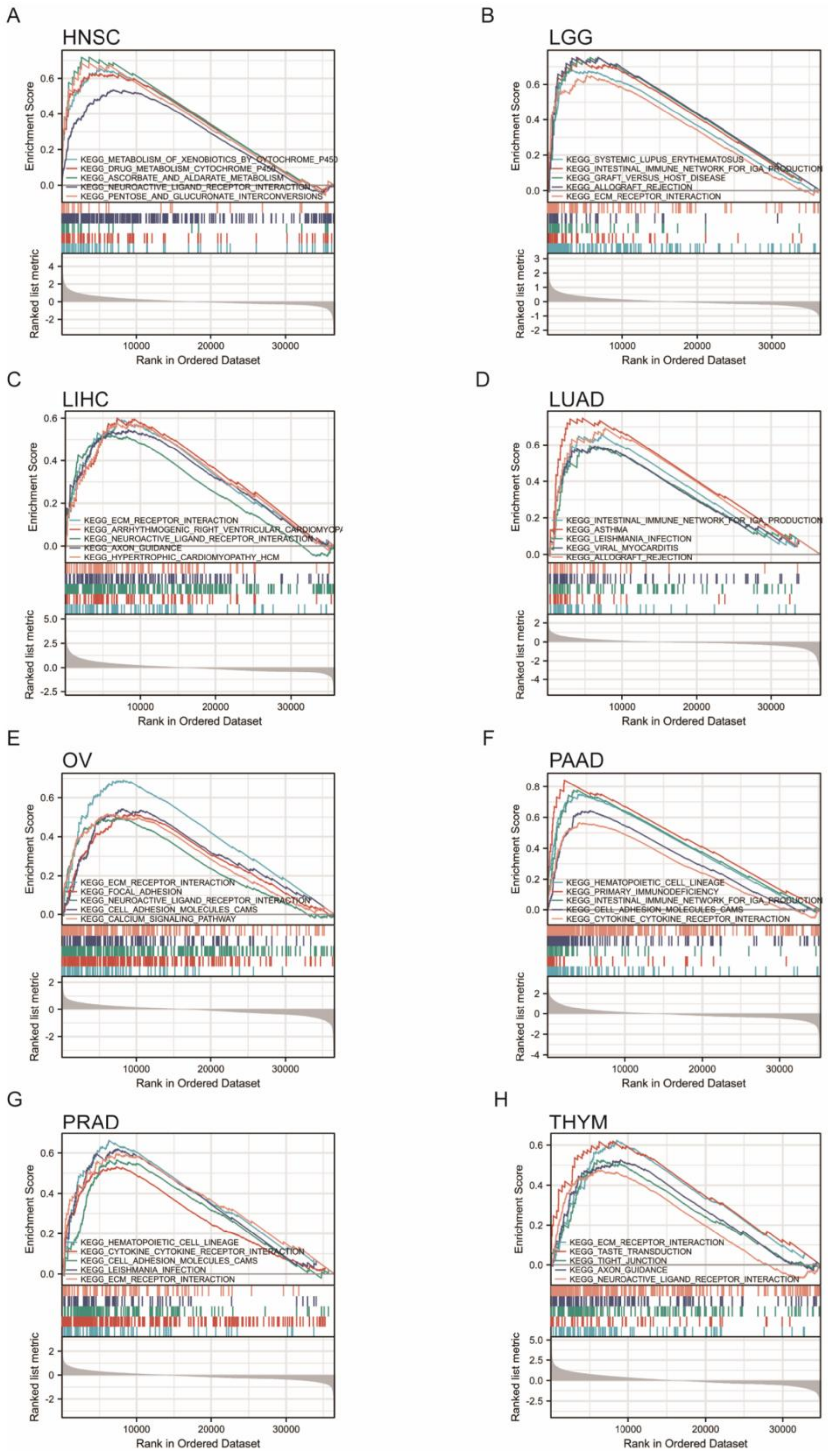

Supplementary Figure S8. GSEA was performed to explore the KEGG signatures of GGA2 in multiple cancer types, including HNSC (A), LGG (B), LIHC (C), LUAD (D), OV (E), PAAD (F), PRAD (G), and THYM (H). Different colored curves are utilized to represent various functions or pathways. The peak of the upward - sloping curve denotes the positive regulation of GGA2 within a particular function or pathway. Conversely, the peak of the downward - sloping curve represents the negative regulation of GGA2.
