# Supplementary material for: Exploring the Role of GGA2 in Cancer Progression: Pan-Cancer Bioinformatics and Experimental Validation in Prostate Cancer
Source: Int J Mol Sci. 2026 Mar 23;27(6):2905. doi: 10.3390/ijms27062905 (PMC13026977; doi:10.3390/ijms27062905)
Supplement: Supplementary file 1 [file ijms-27-02905-s001.zip › Supplementary Tables.pdf]

Supplementary Table S1: Major Core Databases and Their Access Links

Core Database	URL
TCGA	https://portal.gdc.cancer.gov/
GTEx	https://gtexportal.org/home/
UCSCXena	https://xenabrowser.net/
Kaplan–Meierplotter	https://kmplot.com/analysis/
GEPIA2.0	http://gepia2.cancer-pku.cn/
cBioPortal	https://www.cbioportal.org/
UALCAN	http://ualcan.path.uab.edu/
TIDE	http://tide.dfci.harvard.edu/
OncoSplicing	https://oncsplicing.com/
TIMER2.0	http://timer.cistrome.org/
TISIDB	http://cis.hku.hk/TISIDB/
SpatialDB	https://ngdc.cncb.ac.cn/crost/
TISCH	https://tisch.comp-genomics.org/
GeneMANIA	https://genemania.org/
StarBasev2.0	http://starbase.sysu.edu.cn/
cMap	https://portals.broadinstitute.org/cmap/
CellMiner	https://discover.nci.nih.gov/cellminer/
PDB	https://www.rcsb.org/
PubChem	https://pubchem.ncbi.nlm.nih.gov/
HPA	https://www.proteinatlas.org/
CCLE	https://portals.broadinstitute.org/ccle/
SangerBox	https://sangerbox.com/
MicroBioinfo	https://www.microbioinfo.cn/

Supplementary Table S2 Comparison of GGA2 mRNA levels between the control group and the GGA2-OE group （n=3, $\bar{x} \pm s$）

Group	GGA2/β-actin	P-value
Ctrl	1.000±0.004356	
OE-GGA2	12.86±0.009717	＜0.0001

Supplementary Table S3 Comparison of GGA2 protein levels between the control group and the GGA2-OE group （n=3, $\bar{x} \pm s$）

Group	GGA2/β-actin	P-value
Ctrl	0.9838±0.04183	
OE-GGA2	2.573±0.6346	0.0124

Supplementary Table S4 Comparison of GGA2 clone formation ability between the control group and the GGA2-OE group（n=3, $\bar{x} \pm s$）

Group	GGA2/β-actin	P-value
Ctrl	1.000±0.1197	
OE-GGA2	0.4476±0.03637	0.0016

Supplementary Table S5 Comparison of GGA2 cell viability between the control group and the GGA2-OE group（n=3, $\bar{x} \pm s$）

Group	GGA2/β-actin	P-value
Ctrl	100.0±17.63	
OE-GGA2	70.50±14.66	0.0421

Supplementary Table S6 Comparison of GGA2 migration ability between the control group and the GGA2-OE group（n=3, $\bar{x} \pm s$）

Group	GGA2/β-actin	P-value
Ctrl	65.67±5.508	
OE-GGA2	47.33±5.774	0.0164

Supplementary Table S7 Comparison of GGA2 invasion ability between the control group and the GGA2-OE group（n=3, $\bar{x} \pm s$）

Group	GGA2/β-actin	P-value
Ctrl	1.000±0.08206	
OE-GGA2	0.4643±0.03674	0.0005
