# Supplementary figures and images for: Exploring the Role of GGA2 in Cancer Progression: Pan-Cancer Bioinformatics and Experimental Validation in Prostate Cancer
Source: Int J Mol Sci. 2026 Mar 23;27(6):2905. doi: 10.3390/ijms27062905 (PMC13026977; doi:10.3390/ijms27062905)

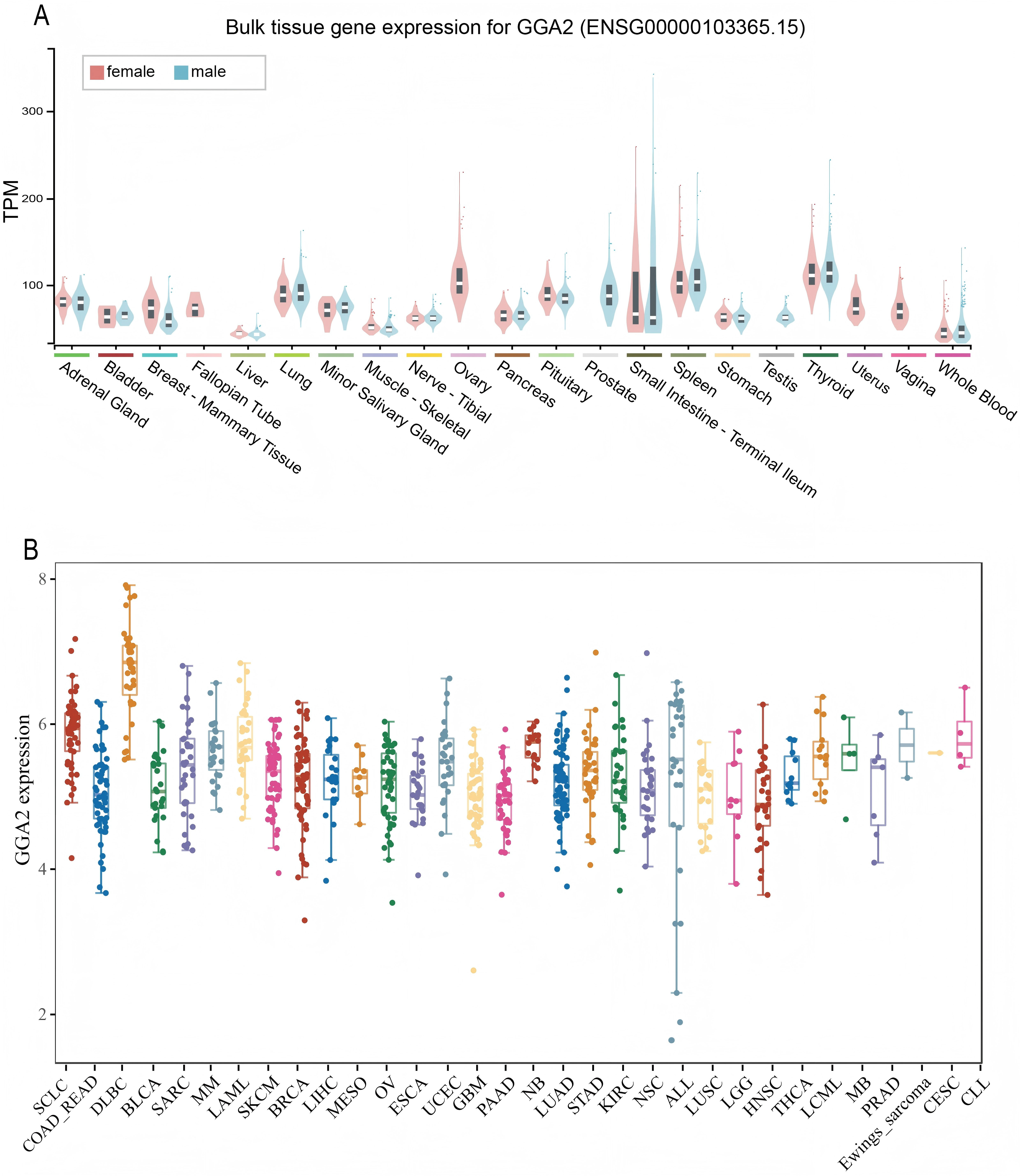

Supplement: Supplementary file 1 [file ijms-27-02905-s001.zip › Figure S1.jpg]

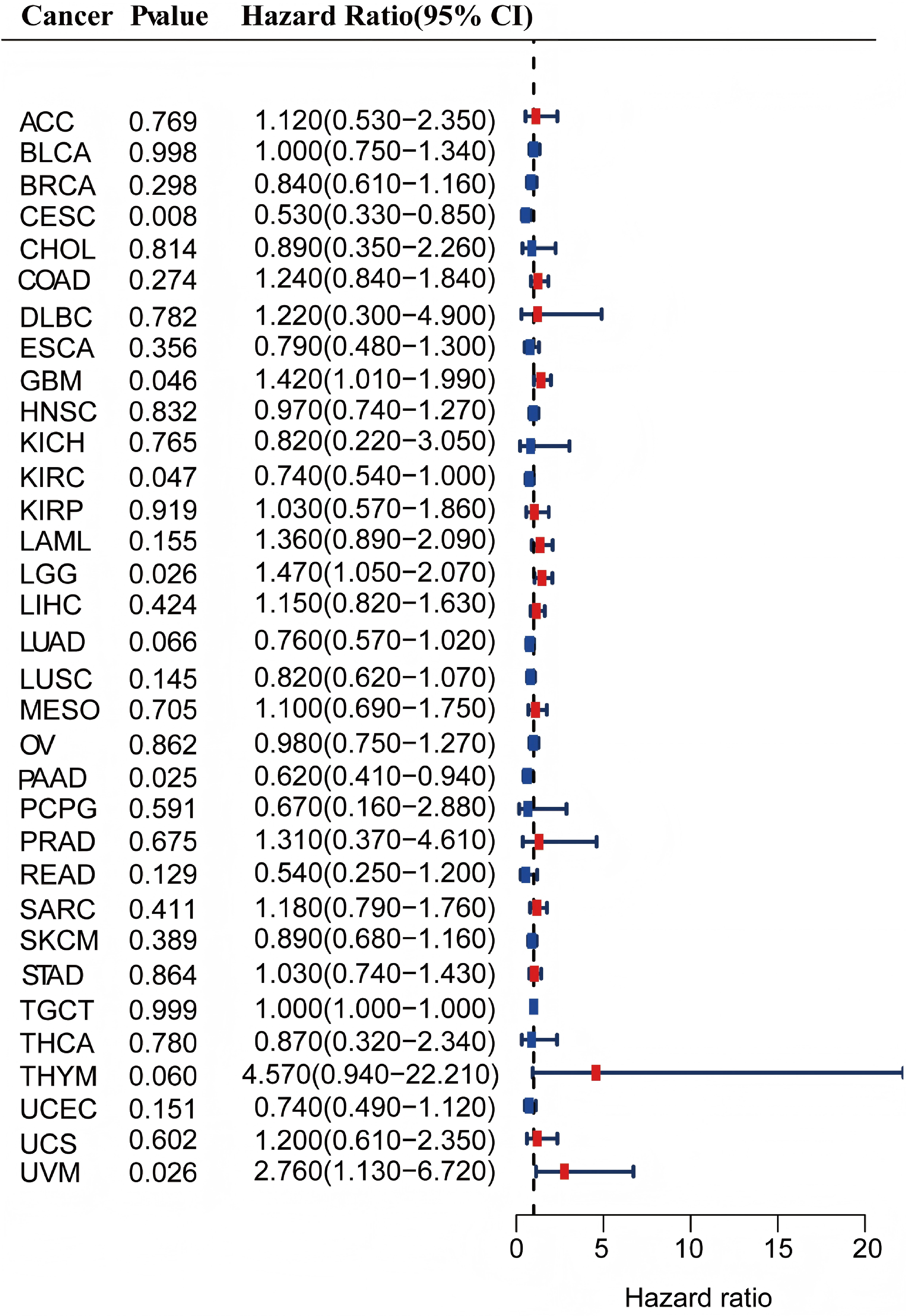

Supplement: Supplementary file 1 [file ijms-27-02905-s001.zip › Figure S2.jpg]

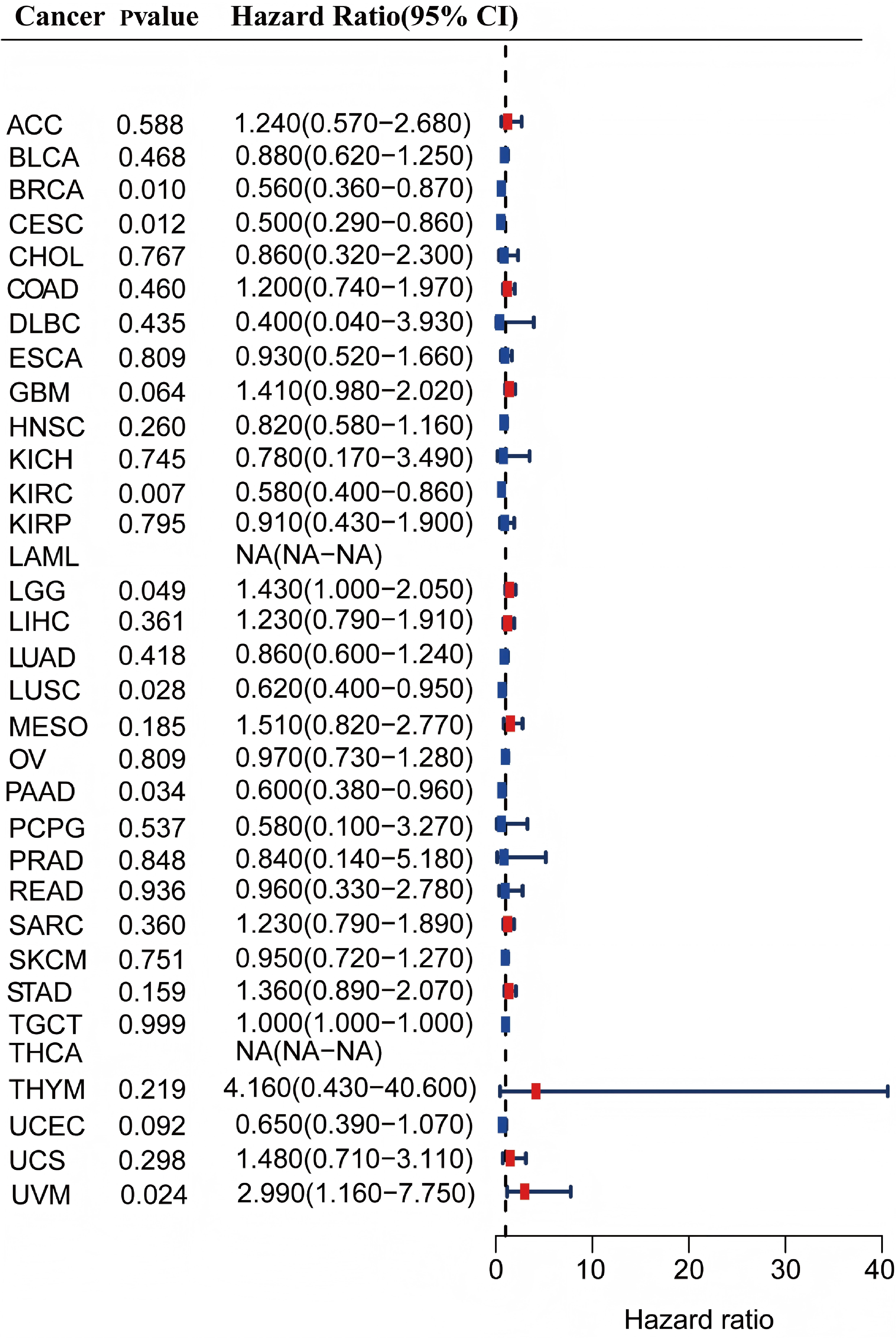

Supplement: Supplementary file 1 [file ijms-27-02905-s001.zip › Figure S3.jpg]

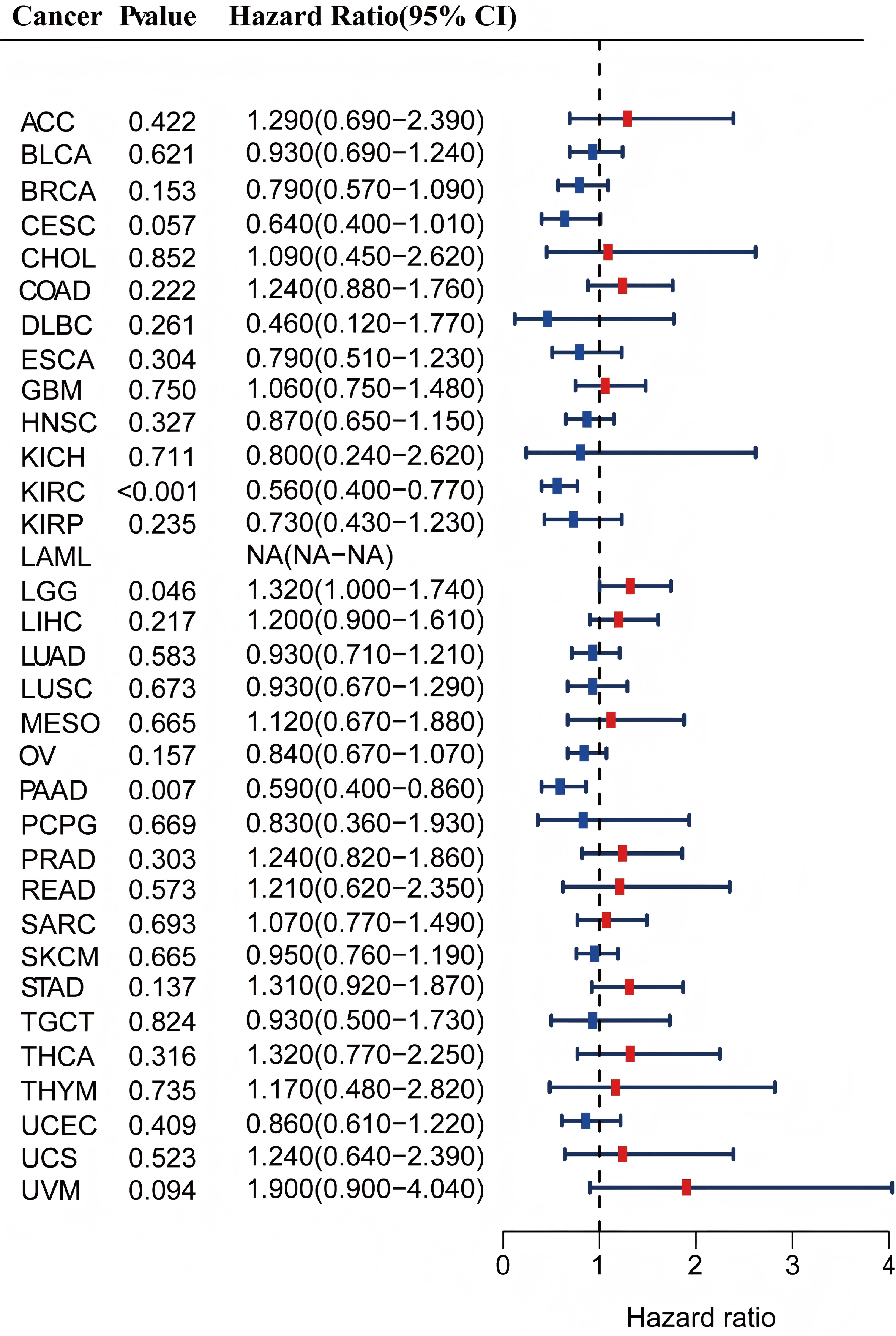

Supplement: Supplementary file 1 [file ijms-27-02905-s001.zip › Figure S4.jpg]

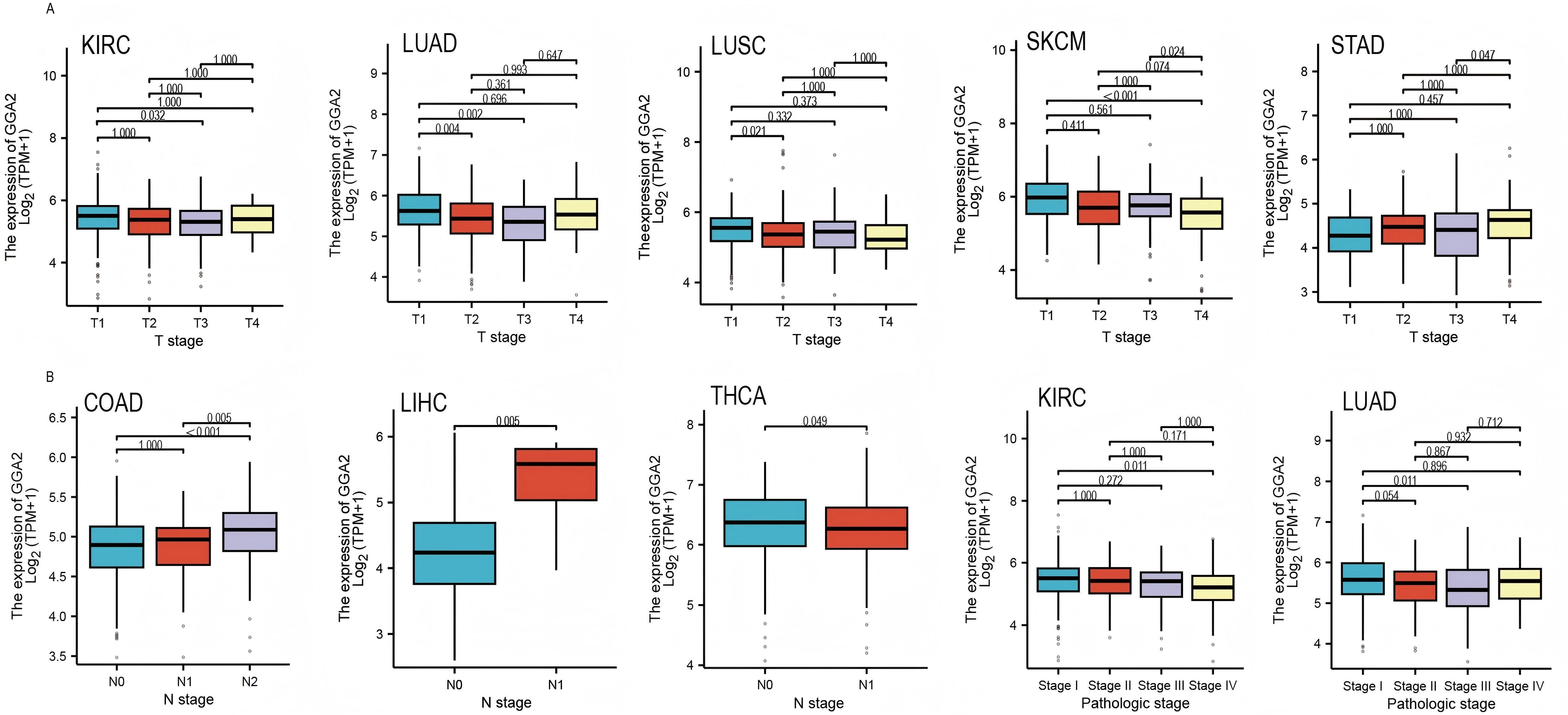

Supplement: Supplementary file 1 [file ijms-27-02905-s001.zip › Figure S5.jpg]

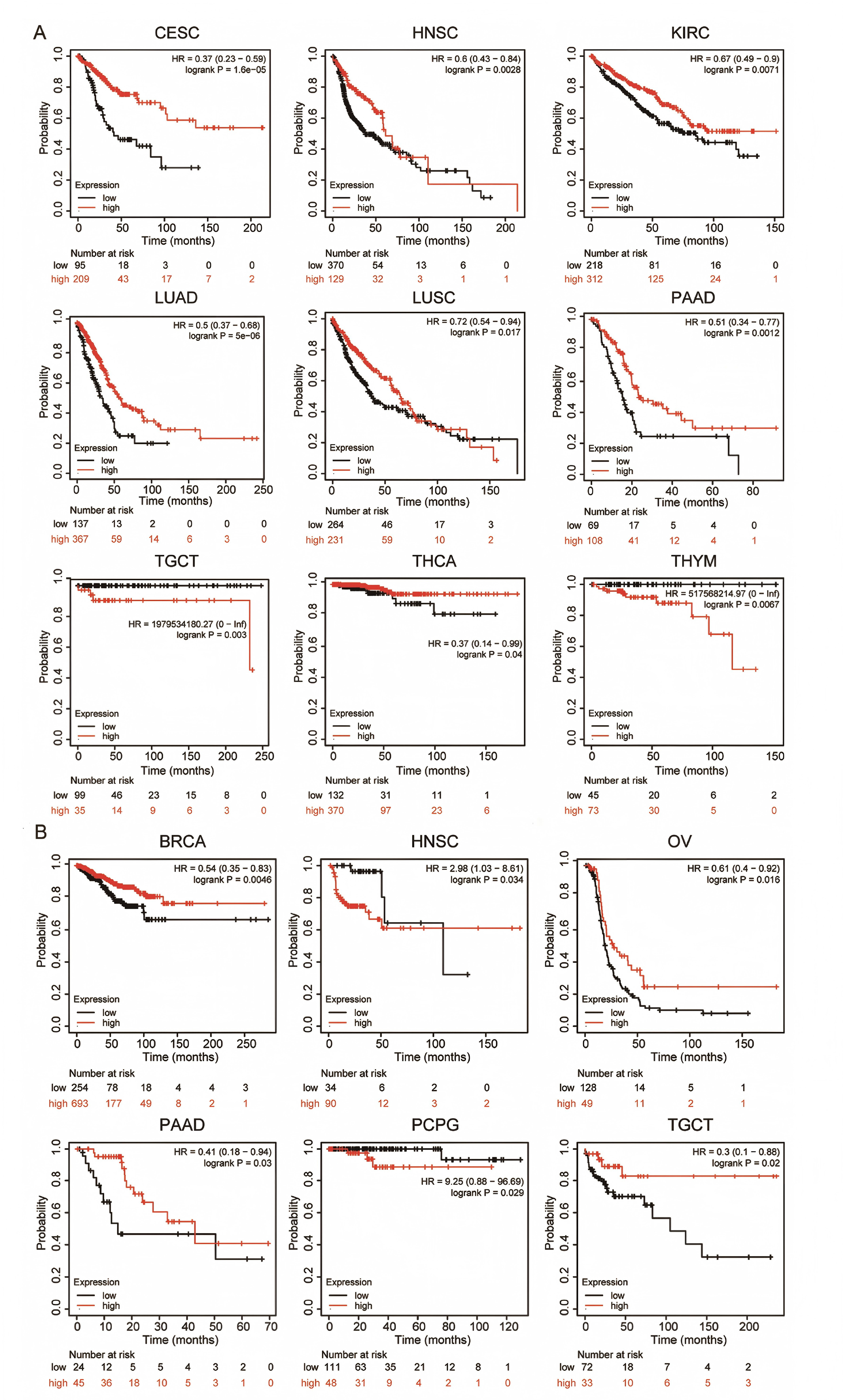

Supplement: Supplementary file 1 [file ijms-27-02905-s001.zip › Figure S6.jpg]

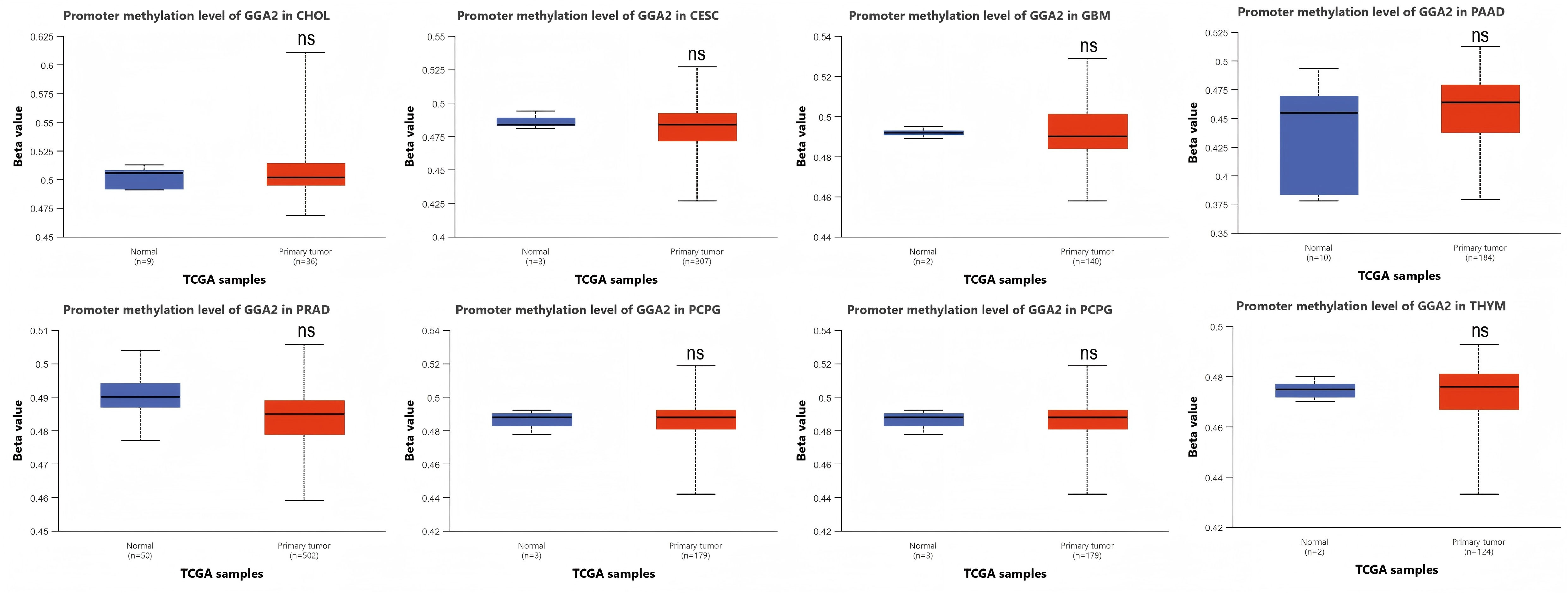

Supplement: Supplementary file 1 [file ijms-27-02905-s001.zip › Figure S7.jpg]

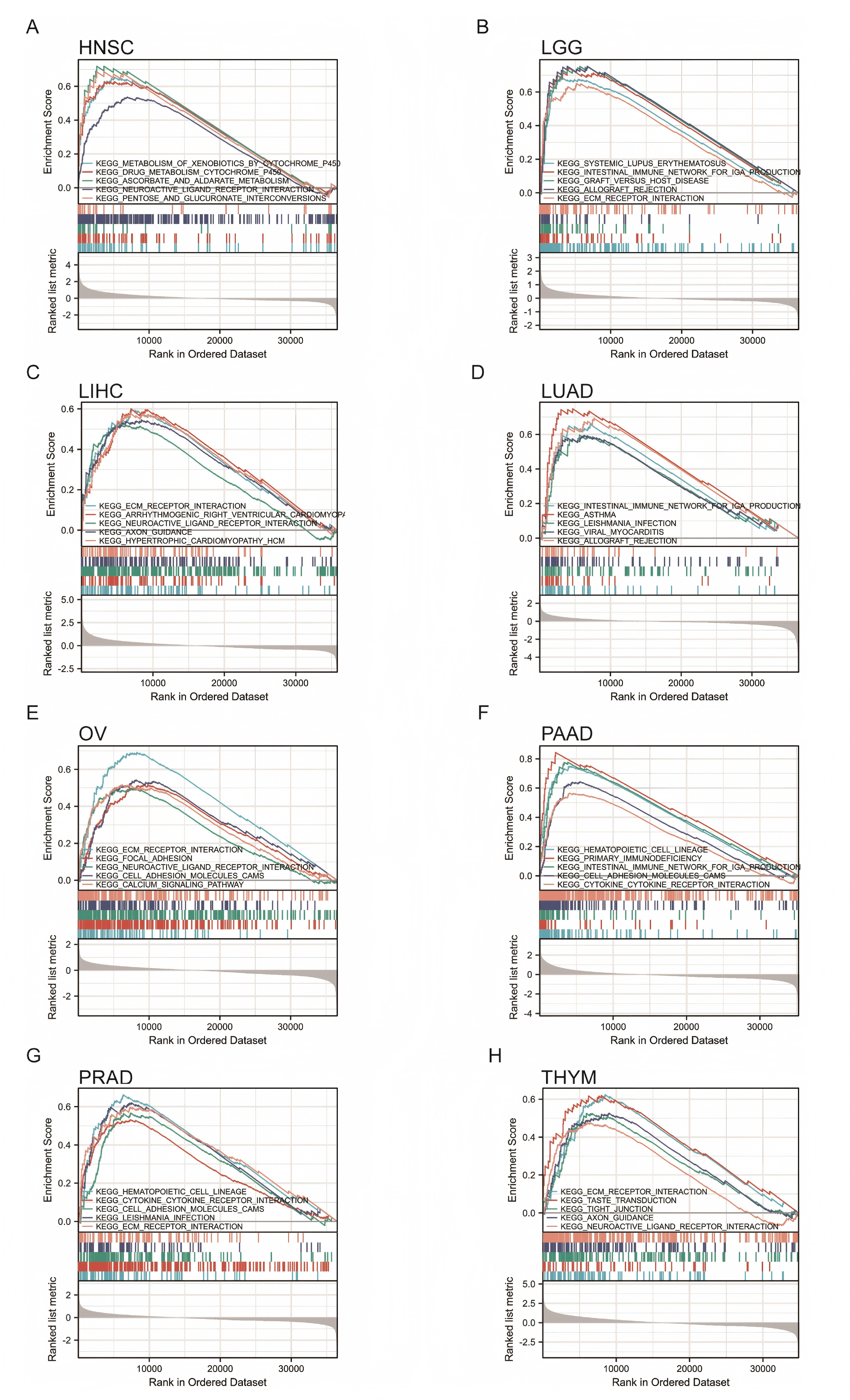

Supplement: Supplementary file 1 [file ijms-27-02905-s001.zip › Figure S8.jpg]
